# Lyso-Lipid-Induced Oligodendrocyte Maturation Underlies Restoration of Optic Nerve Function

**DOI:** 10.1523/ENEURO.0429-21.2022

**Published:** 2022-01-24

**Authors:** Anddre Osmar Valdivia, Sanjoy K. Bhattacharya

**Affiliations:** 1 Department of Ophthalmology, Bascom Palmer Eye Institute, University of Miami, Miami, Florida 33136; 2 Neuroscience Graduate Program, University of Miami, Miami, Florida 33136

**Keywords:** deimination, LPC 18:1, myelin, oligodendrocytes, optic nerve, remyelination

## Abstract

Protein hyperdeimination and deficiency of lyso-phospholipids (LPC 18:1) has been associated with the pathology of demyelinating disease in both humans and mice. We uncovered interesting biology of LPC 18:1, in which LPC 18:1 induced optic nerve function restoration through oligodendrocyte maturation and remyelination in mouse model systems. Our *in vitro* studies show LPC 18:1 protection against neuron-ectopic hyperdeimination and stimulation of oligodendrocyte maturation, while *in vivo* investigations recorded optic nerve function improvement following optic nerve injections of LPC 18:1, in contrast with LPC 18:0. Thus, just a change in a single bond renders a dramatic alternation in biological function. The incorporation of isobaric C13-histidine in newly synthesized myelin proteins and quantitative proteome shifts are consistent with remyelination underlying restoration in optic nerve function. These results suggest that exogenous LPC 18:1 may provide a therapeutic avenue for stemming vision loss in demyelinating diseases.

## Significance Statement

Demyelinating diseases have been associated with an increase in aberrant hyperdeimination and deficiencies in neuronal lipids (LPC 18:1). We demonstrate that exogenous delivery of LPC 18:1 can prevent hyperdeimination and restore optic nerve function in demyelinating optic nerves. We show that this effect is mediated by the induction of oligodendrocyte maturation and remyelination. These effects contrast the demyelinating effects of LPC 18:0, which only differs by a single bond, pointing toward a fundamental change in our understanding of lipid behavior. Our findings highlight the potential translational implications of LPC 18:1 in restoring optic nerve function in demyelinating diseases.

## Introduction

Demyelinating diseases [e.g., multiple sclerosis (MS)] are often associated with loss of visual function, because of progressive demyelination of the optic nerve (Park et al., 2020; Lee et al., 2021; Murphy et al., 2021). Visual function loss, once thought to be transient, has now been firmly established as an irreversible paradigm of demyelinating diseases by objective electrophysiological measurements of inner retinal function (Boquete et al., 2019; Ziccardi et al., 2020; Jiang et al., 2021). Heterogeneous patchy demyelination and consequent vision loss are likely to occur in other broad neurodegenerative ocular diseases including glaucoma and various forms of optic neuritis (Klistorner et al., 2007). Despite heterogeneous clinical manifestations, active demyelinating lesions share classical molecular features that include loss of myelin proteins, aberrant hyperdeimination, and deficiencies in lipid composition (Göpfert et al., 1980; Whitaker et al., 1992; Moscarello et al., 1994; Wood et al., 1996; Raijmakers et al., 2005; Bhattacharya et al., 2008; Breij et al., 2008; Bhattacharya, 2009; Del Boccio et al., 2011; Valdivia et al., 2020).

Hyperdeimination was discovered to positively correlate with severity of MS >30 years ago (Whitaker et al., 1992; Raijmakers et al., 2005). Deimination or citrullination of protein-bound arginine is a post-translational modification that neutralizes the positive charge in arginine (Vossenaar et al., 2003). It was established that neuron-ectopic hyperdeimination is part of the pathology in MS and increases with disease severity (Moscarello et al., 1994; Wood et al., 1996; Bhattacharya et al., 2008; Bhattacharya, 2009; Valdivia et al., 2020). Myelin basic protein isoform 5 (MBP5) is normally deiminated only at 6 sites in a distributive manner but undergoes aberrant deimination in other 13 sites during demyelination (Moscarello et al., 1994; Zhou, et al., 1995; Wood et al., 1996; Cao et al., 1999; Valdivia et al., 2020). Loss of charge because of deimination disrupts protein structure, which increases susceptibility to proteolysis, generating immunogenic peptides that trigger an autoimmune response (Musse et al., 2006). A lack of lipid complexation with MBP5 has also been documented to result in vulnerability of deimination by peptidyl arginine deiminases (PADs), therefore highlighting the importance of protein–lipid interactions for maintenance of normal myelination (Beniac et al., 1997; Ridsdale et al., 1997; Haas et al., 2004; Valdivia et al., 2020). During *in vitro* conditions, PADs and PAD2 (the major deiminase in the CNS) behave as processive enzymes, findings that have been found to be consistent *in vivo* (Vossenaar et al., 2003).

Lack of lipid complexation with myelin proteins is an initial event that precludes selective distributive assignment of normal deimination patterns. Only 6 arginines of a total of 19 are deiminated in MBP5 in healthy individuals, contrasting the progressive deimination of additional sites observed in demyelinating diseases. Our initial *in vitro* experiments with model systems are consistent with this hypothesis and identified a lyso-phospholipid, LPC 18:1, as a candidate lipid that can protect against aberrant hyperdeimination (Valdivia et al., 2020). To test the potential translational implications LPC 18:1 has on optic nerve function, we tested its biological effects *in vitro* and *in vivo*, while contrasting with LPC 18:0 (a known demyelinating agent that differs structurally only in a single bond).

## Materials and Methods

### Generation of experimental autoimmune encephalomyelitis models

All animals were housed at the McKnight vivarium (University of Miami, Miami, FL), and animals used in this study were under the University of Miami Institutional Animal Care and Use Committee-approved protocols (protocol no. 16–235 and no. 19–194). The method was adapted from our previous study (Valdivia et al., 2020). Immunizing neuroantigen was prepared by an emulsion consisting of 2 mg of myelin oligodendrocyte glycoprotein [MOG (i.e., MOG35-55); catalog #12668, Biosynthesis] or myelin proteolipid protein (PLP139-151; catalog #13352, Biosynthesis), incomplete Freund’s adjuvant (catalog #90003–748, VWR), and 6 mg of *Mycobacterium tuberculosis* (H37RA; catalog #DF3114-33–8, Thermo Fisher Scientific). This stock solution was used to immunize six animals, in which each individual animal received 0.333 mg of MOG35-55 or PLP139-151 and 1 mg of *M. tuberculosis*. Pertussis toxin (catalog #181, Biological Laboratories) was prepared at a concentration of 50 μg/ml in sterile PBS, pH 7.4, with intraperitoneal administration of 200 ng/injection. Female C57BL/6J or SJL/J mice were injected intraperitoneally with pertussis toxin the day before injection. They were then immunized subcutaneously at 2 months of age with 200 μl of neuroantigen (MOG35-55 or PLP139-151) followed by an intraperitoneal injection of pertussis toxin 2 d postimmunization. The following four cohorts were created for this study: an immunized group with MOG35-55, an immunized group without MOG35-55 (sham injection control), an injection control that received no injections [wild-type (WT) control], and an immunized group with PLP139-151. All C57BL/6J animals were immunized with MOG35-55, and all SJL/J animals were immunized with PLP139-151. Animals were monitored for decline in body weight as well as manifestation of clinical signs indicative of encephalomyelitis following the same criteria as the previous study (Valdivia et al., 2020).

### Gel electrophoresis and immunoblotting

Total protein was assessed using the Pierce BCA Protein Assay Kit (catalog #23225, Thermo Fisher Scientific) following manufacturer recommendations. Samples were resuspended in Tris-glycine-SDS at equal concentrations based on total protein assay and heated to 85°C for 3 min. Samples were then diluted to working concentration using Laemmli loading buffer, as indicated by the manufacturer (catalog #M337, Amresco). Samples were loaded in 4–20% Tris-glycine gel (catalog #5671095, BIO-RAD) and ran using Tris-glycine-SDS running buffer at 120 V, with a cap at 360 A for 60 min. PVDF membrane was activated with methanol for 1 min, washed multiple times with ultrapure water and washed with transfer buffer (TGS-20% methanol) before transfer. Semidry transfer was conducted using the Trans-Blot Turbo Transfer System (catalog #1704150, BIO-RAD) following manufacturer recommendations. Membrane was blocked with blocking buffer (catalog #170–6404, BIO-RAD) and probed with primary antibodies at 1:500 dilution. Membrane was washed using wash buffer (25 mm Tris, 2.6 mm KCl, 0.14 m NaCl, 0.2% Tween 20, at pH 8) and probed with secondary antibodies at 1:1000 dilution. Membrane was incubated with chemiluminescence Western blotting substrate (catalog #32106, Thermo Fisher Scientific) following manufacturer recommendations and was imaged using the ImageQuant LAS 4000 (catalog #08855–1327, GE Healthcare). Dot immunoblotting was performed as described above with the exception that samples were not separated by gel electrophoresis. A modification to this protocol was made when detecting total deimination by modification of the citrulline residues through a reaction with 2,3-butanedione monoxime and antipyrine in a strong acid solution (catalog #17-347B, Millipore) following manufacturer recommendations, which was performed immediately after transfer, and the modification was incubated at 37°C for 3 h. Densitometry analysis was done using the ImageJ program (National Institutes of Health).

### Antibodies

The following antibodies were used: MBP (catalog #ab7349, Abcam); citrulline (Cit; catalog #MABS54887, Millipore); glyceraldehyde-3-phosphate dehydrogenase (catalog #ab22556, Abcam); platelet-derived growth factor receptor α (PDGFR-α; ab134123, Abcam); MOG (catalog #ab28766, Abcam); β-actin (catalog #ab8226, Abcam); oligodendrocyte surface protein (OSP; catalog #ab7474, Abcam); sorting nexin 4 (SNX4; catalog #ab198504, Abcam); peroxisome proliferator-activated receptor-δ (PPAR-δ; catalog #ab23673, Abcam); chromodomain helicase DNA binding protein 7 (CHD7; catalog #6505S, Cell Signaling Technology); and protein piccolo (PCLO; catalog #ab20664, Abcam).

### MBP5 extraction

BL21 competent cells (catalog #L1195, Promega) were transformed using a standard protocol with either a mouse MBP5 construct or a human MBP5 construct (catalog #EX-Mm03796-B10 and #EX-D0136-B10, respectively, GeneCopoeia). In brief, cells were thawed on ice and incubated on ice for 30 min with 5 ng of DNA. A heat shock at 42°C was given in a water bath for 20 s, followed by incubation on ice for 2 min. Cells were supplemented with SOC media and incubated at 37°C for 1 h at 225 rpm. Ampicillin LB plates were plated with 5 and 10 μl of transformed cells and incubated overnight at 37°C. Glycerol stocks were made from the remaining cells and stored at −80°C. Five milliliters of starter culture in ampicillin LB broth (100 μg/ml ampicillin) were inoculated with one bacterial colony and incubated at 37°C overnight at 225 rpm. Two hundred fifty milliliters of ampicillin LB broth was inoculated with 5 ml of starter culture and incubated at 37°C at 225 rpm until culture reached OD of 0.5–0.6. Culture was cooled down to room temperature, supplemented with 1 mm IPTG (Isopropyl β-D-1-thiogalactopyranoside), and induced expression overnight at room temperature. Cells were collected by centrifugation at 3500 × *g* for 20 min, washed with PBS, and recentrifuged. Cells were lysed by repeated freeze–thaw cycles while resuspended in hypotonic water. Following the last freeze–thaw cycle, protease inhibitors, concentrated PBS and Dnase, were added to lysate. Lysates were incubated for 5 min at 37°C then centrifuged at 3000 × *g* for 20 min, the supernatant was transferred to a new tube, and the pellet was discarded.

### Immunoprecipitation

Immunoprecipitation methods were adapted from our previous study (Ding et al., 2019). Lysates prepared from bacteria were subjected to immunoprecipitation using an antibody for MPB (catalog #ab7349, Abcam). Briefly, immunoprecipitation was conducted as follows: ∼67 μg of Sepharose B beads were suspended overnight in 200 μl of 50 mm sodium borate buffer, pH 9.0, and were incubated with 10 μg of antibodies at room temperature for 1 h. The beads and antibody were cross-linked by adding 10 μg of dimethyl pimelimidate dihydrochloride three times each with 2 h incubation at room temperature and then were kept at 4°C overnight. Antibody-coupled beads were subsequently neutralized with 200 μl of 200 mm ethanolamine and washed with 1 ml of PBS twice. The antibody-coupled beads were then incubated with 200 μg of lysate for 1 h at room temperature. The beads were washed twice with 500 μl of PBS and eluted with two 20 μl volumes of 100 mm glycine, pH 3.0. The eluents were combined and divided into two equal amounts. Confirmation of the presence of MBP was done via dot immunoblot as described above.

### MBP-lipid deimination assay

Thirty micrograms of either LPC 18:1 or LPC 18:0 was incubated in the presence of 10 μg of recombinant MBP protein in PBS and allowed to interact for 30 min at room temperature. After incubation, each reaction was adjusted to a final concentration of 1.66 mm CaCl2 and incubated overnight at 37°C in the presence of purified PAD (catalog #P1584, Sigma-Aldrich). Proteins were then precipitated using standard acetone precipitation protocols, evaporated using the CentriVap Concentrator system (catalog #64132–2696, Labconco), and processed for mass spectrometry analysis (see section Sample preparation for protein mass spectrometry).

### Lipid raft isolation

Lipid rafts isolation protocol was adapted from a previous study (Persaud-Sawin et al., 2009). In brief, neuronal tissue was homogenized in detergent-free lysis buffer at 4°C (TBS pH 8; PMSF 1 mm; sodium orthovanadate 1 mm; CaCl2 1 mm; MgCl2 1 mm; protease inhibitor cocktail, catalog #A32963, Thermo Fisher Scientific) and sheared through a 23 gauge needle. Homogenate was centrifuged at 1000 × *g* for 10 min at 4°C, and the supernatant was collected in a separate tube. The pellet was resuspended in detergent-free lysis buffer and sheared through a 23 gauge needle, then centrifuged at 1000 × *g* for 10 min at 4°C. Supernatant was collected and added to the previous collection. The sample was store at −80°C or immediately used in sucrose density centrifugation. Sample was resuspended in sucrose for a final 42.5% (w/v). A layer of 36% (w/v) sucrose was added on top followed by a 5% (w/v) sucrose layer. Sucrose layers were then centrifuged at 200,000 × *g* for 18 h at 4°C. Eighteen sequential fractions were collected and subsequently immunoblotted for lipid raft markers (Flot1 and TfR). Fractions were also probed for total cholesterol content using the Total Cholesterol Assay Kit (catalog #STA-384, Cell Biolabs) following manufacturer recommendations. Total protein was assessed using the Pierce BCA Protein Assay Kit (catalog #23225, Thermo Fisher Scientific) following manufacturer recommendations. Fractions had to test positive for Flot1 (catalog #ab78178, Abcam), negative for TfR (catalog #ab84036, Abcam), have low protein content and high cholesterol content for meeting lipid raft criteria.

### Optic nerve injections

Animals were anesthetized by intraperitoneal injection of 100 μl of a ketamine and xylazine cocktail (1.5 mg/0.3 mg per 100 μl) per 20 g of body weight. Animals were given a toe pinch before beginning the procedure to ensure deep anesthesia. A drop of balanced saline solution was delivered to the eye to prevent dryness, and any excess was wiped using a sponge tip applicator. Using forceps (Student Dumont #7, catalog #91197–00, and Dumont Forceps-Micro-Blunted Tips, catalog #11253–20, Fine Science Tools), the conjunctiva was lifted, and a small incision was made with spring scissors [Vannas Spring Scissors (2.5 mm cutting edge); catalog #15001–08, Fine Science Tools]. This incision was enlarged to allow access to the optic nerve. Forceps were inserted between the superior rectus and lateral rectus muscles and were pried open by allowing forceps to gradually return to their open position. While holding this position, the optic nerve was fully exposed and a small incision in the meninges was made with a pointed-end 26 gauge (26G) needle (PrecisionGlide Needle; catalog #305111, BD). This incision was used to insert a blunt-end 33 gauge (33G) Hamilton needle (catalog #7803 -05, Hamilton; 5 μl Microliter Syringe, model 65 RN, catalog #7633 -01, Hamilton) and deliver 0.5 μl of lipid mixture (LPC 18:1 at 150 μm or LPC 18:0 at 150 μm or LPC 18:1 at 150 μm + C13-His at 10 μm). The eye was then left to recover and a neomycin/polymyxin B sulfate/dexamethasone ophthalmic ointment (catalog #80214–942, Sandoz) was applied to the eye. Animals were kept in a 37°C warm pad after the procedure and monitored for full anesthesia recovery. Animals were returned to their cages, and food pellets were placed at ground level along with hydration packs for easy access. Animals were monitored daily for any signs of health decline and eye infections.

### Electroretinogram recordings

Pattern electroretinogram (PERG) and flash electroretinogram (FERG) was recorded using the Jörvec PERG system was used (PERG Visual Stimulation Box; catalog #M014760L, Jörvec). The experimental setup for FERG was as described in previously published protocols; however, there was a slight modification for the experimental setup for PERG (Valdivia et al., 2020). The modification consisted of the subcutaneous insertion of the recording electrode between the two eyes, the reference electrode in the scalp, and the ground electrode in the lower back adjacent to the tail. For both PERG and FERG, animals were anesthetized by intraperitoneal injection of 100 μl of a ketamine and xylazine cocktail (1.5 mg/0.3 mg per 100 μl) per 20 g of body weight. A drop of balanced saline solution was delivered to the eye to prevent dryness for the duration of the procedure. Animals were situated in a heating pad (model TCAT-2LV Controller, Physitemp) with an anal reference thermometer set at 37°C to stabilize internal body temperature. PERG was recorded by three consecutive responses of 600 contrast reversals, and recording settings consisted of 10.0 K gain, 1.0 Hz high pass, 100.0 Hz low pass, and 360.0 μV rejection. FERG was recorded by three consecutive measurements of a flash with a strength of 20.0 candela second per square meter (cd*s/m^2^) and a frequency of 1.0 Hz. The amplitudes for PERG and FERG were measured as the difference (in microvolts) between the highest peak and the consecutive lowest trough. Latency values for PERG and FERG were measured as the time (in milliseconds) it took to reach the highest peak (from time 0 to time when highest peak was recorded).

### Immunohistochemistry

Dissected optic nerves were immersed fixed in 4% paraformaldehyde in PBS and incubated overnight at 4°C. Tissue was washed using PBS followed by cryoprotection using a gradient of 10% sucrose (overnight at 4°C), 20% sucrose (overnight at 4°C), and 30% sucrose (overnight at 4°C). Tissue was then embedded in optimal cutting temperature compound (catalog #25608–930, VWR) and stored at −80°C. Sections were collected at 10 μm thickness and stored at −80°C. A standard immunohistochemistry protocol was used to detect MBP (catalog #ab7349, Abcam), CD90 (catalog #CBL1354, CYMBUS Biotech), and tissue was mounted using Vectashield with DAPI (catalog #H-1200, Vector Laboratories).

### Electron microscopy

Samples were fixed in 2% glutaraldehyde in 0.05 m phosphate buffer and 100 mm sucrose, postfixed overnight in 1% osmium tetroxide in 0.1 m phosphate buffer, dehydrated through a series of graded ethanols, and embedded in a mixture of EM-bed/Araldite (Electron Microscopy Sciences). The 1-μm-thick sections were stained with Richardson’s stain for observation under a light microscope. One hundred nanometer sections were cut using a Leica Ultracut-R ultramicrotome and were stained with uranyl acetate and lead citrate. The grids were viewed at 80 kV on a transmission electron microscope (model JEM-1400, JEOL), and images were captured with a digital camera (BioSprint, AMT).

### Sample preparation for mass spectrometry

Fifteen micrograms of total protein from homogenate sample were added to four times the volume of acetone at 20°C and incubated at 20°C overnight. Samples were centrifuged at 21,000 × *g* at 4°C for 30 min (Megafuge 8R, Thermo Fisher Scientific). The supernatant was discarded, and the pellet (very small) was air dried for 10 min. The pellet was resuspended, denatured, and reduced with 6 m urea and 10 mm dithiothreitol in 50 mm ammonium bicarbonate for 1 h at room temperature. Following denaturing and reduction, the resuspended pellet was alkylated with 15 mm iodoacetamide in 50 mm ammonium bicarbonate for 30 min while maintained in darkness. Alkylation reaction was quenched using 20 mm dithiothreitol in 50 mm ammonium bicarbonate for 1 h at room temperature while kept in darkness. The sample was diluted using 50 mm ammonium bicarbonate to contain 1 m urea. The sample was digested using either trypsin (catalog #V5111, Promega) or chymotrypsin (catalog #V106A, Promega) at a 1:30 (w/w) ratio of enzyme to protein. Digestion was incubated overnight at 37°C. Reaction was terminated using 50% formic acid at a 5:100 (v/v) ratio of formic acid to sample volume. Samples were stored at −20°C or immediately desalted. Samples were desalted using the Pierce Graphite Spin Columns (catalog #88302, Thermo Fisher Scientific) following manufacturer recommendations. Samples were then evaporated using the CentriVap Concentrator System (catalog #64132-2696, Labconco) and resuspended in 30 μl of protein resuspension solution [2% (v/v) acetonitrile, 0.1% (v/v) formic acid in mass spectrometry-grade water]. Lipids were harvested by adding 400 μl of a 1:1 (v/v) methanol/chloroform mix to 200 μl of cell culture lysate. Lysate was vortexed and incubated in ice for 5 min, followed by the addition of 350 μl of chloroform. Lysate was centrifuged at 18,000 × *g* for 15 min at 4°C. The organic layer containing lipids (bottom layer) was transferred to a new tube and desiccated using the CentriVap Concentrator System. Lipids were resuspended in 50 μl of lipid resuspension solution [50% (v/v) isopropyl alcohol and 50% (v/v) acetonitrile].

### High-performance liquid chromatography-mass spectrometry

Mass spectrometry lipidomic chromatography and identification were conducted as described in detail in our previous publication (Chauhan et al., 2019). In brief, samples were run through an Acclaim C30 column (particle size, 3.0 μm; inner diameter, 150 × 2.1 mm; Thermo Fisher Scientific). An HPLC ACCELA instrument (equipped with an autosampler and 600 LC pump, Thermo Fisher Scientific) was coupled to a Q Exactive Orbitrap Mass Spectrometer (Thermo Fisher Scientific). Heated electrospray ionization (HESI) was used as the method of ionization by coupling a HESI probe to the Q Exactive instrument. The instrument was set at full scan with collision energy of 30 and 19 eV in positive mode. Lipids were identified using LipidSearch software version 4.1 (developed by Ryo Taguchi and Mitsui Knowledge Industry Co.). Mass spectrometry proteomics was performed on a Q Exactive instrument after fractionation on a coupled EASY-nLC 1000 Liquid Chromatography System (Thermo Fisher Scientific), as described in our previous published report (Travers et al., 2016). Peaks were generated using Thermo Scientific Xcalibur (version 4.1.31.9, released in 2017), proteins were identified using Proteome Discoverer 2.2 (version 2.2.0.388, released in 2017). The UniProt sequence database was used for the identification of proteins (downloaded July 2019). Proteome Discoverer search parameters for chymotrypsin-digested enzymes were as follows: maximum missed cleavage sites, 2; minimum peptide length, 6; maximum peptide length, 144; modification: deimination, +0.984 Da (R); C13-His, +6.02 Da (H); maximum modifications per peptide, 3; precursor mass tolerance, 10 ppm; fragment mass tolerance, 0.02 Da; signal/noise threshold for spectra, 1.5; false discovery rate (FDR): strict for peptide-spectrum match (PSM), 0.01; strict for peptides, 0.01. In brief, false discovery rates are calculated as follows: first the software ascertains whether there are q-values and posterior error probability (PEP) available for PSMs. If so, the software uses them and assigns the PSM confidences based on target FDRs for PSMs. Next, the software calculates q-values and PEPs for peptides engaging the Qvality algorithm. Peptide confidences are then assigned based on Target FDRs for peptides. If there are no q-values and PEPs available for PSMs, the PSM confidence is set based on our defined target FDRs for PSM using the respective search engine scores.

### Retinal ganglion cell isolation

Primary retinal ganglion cells (RGCs) were isolated according to the two-step immunopanning protocol described in a previous study (Dvoriantchikova et al., 2014). Briefly, whole retinas of 10 postnatal day 11–12 pups were incubated in papain solution (16.5 U/ml; catalog #LS003127, Worthington Biochemical) for 30 min. Macrophages and endothelial cells were removed from the cell suspension by panning with anti-macrophage antiserum (catalog #AIA31240, Accurate Chemical). RGCs were bound to the panning plates containing the antibody against Thy1.2 (catalog #102646–060, VWR) and were then released by incubation with trypsin solution (catalog #T9935, Sigma-Aldrich). RGCs were plated in a plate coated with poly-d-lysine (catalog #P6407, Sigma-Aldrich) and laminin (catalog #L-6274, Sigma-Aldrich). RGCs were not treated (addition of RGC growth media), treated with LPC 18:0 at 10 μm in RGC growth media, or treated with LPC 18:1 at 10 μm in RGC growth media. RGCs were incubated for 24 h and harvested the next day.

### Oligodendrocyte precursor cell culture

Rat oligodendrocyte precursor cells (OPCs) were purchased from ScienCell Research Laboratories (catalog #R160092008) and were cultured according to manufacturer recommendations. In brief, plates were coated with poly-d-lysine (2 μg/cm^2^; catalog #P6407, Sigma-Aldrich) and incubated overnight at 37°C. OPC growth media were prepared using OPC media (catalog #1601, ScienCell Research Laboratories) supplemented with OPC growth supplement (catalog #1652, ScienCell Research Laboratories) and a penicillin/streptomycin solution (catalog #0503, ScienCell Research Laboratories). OPC differentiation medium was prepared using OPC media (catalog #1601, ScienCell Research Laboratories) supplemented with OPC differentiation supplement (catalog #1672, ScienCell Research Laboratories), fetal bovine serum (catalog #0005, ScienCell Research Laboratories), and penicillin/streptomycin solution (catalog #0503, ScienCell Research Laboratories). A poly-d-lysine-coated plate was washed twice with sterile water, OPC growth media were added to each well, and each plate was placed in an incubator to equilibrate for 15 min (at 37°C and 5% CO_2_). The frozen vial containing OPCs was warmed in 37°C water bath and gently rotated to equally suspend cells. A total of 15,000 cells/cm^2^ were seeded and left overnight (∼16 to ∼18 h) in the incubator. Media were changed daily for 2 d. OPCs were not treated (addition of OPC growth media), differentiated (addition of OPC differentiation media), treated with LPC 18:0 at 10 μm in OPC growth media, or treated with LPC 18:1 at 10 μm in OPC growth media. OPCs were incubated for 24 h and harvested the next day. OPCs and oligodendrocytes were further validated using mRNA analysis following published methods (Jäkel et al., 2019).

### siRNA and overexpression

Cell transfection was carried using the INTERFERin transfection kit following manufacturer recommendations (catalog #89129–930, Polyplus-transfection). siRNA for LPCAT1, PLA2G4C, and LIPC was purchased from Ambion (catalog #s102346, #s107315, and #s67780, respectively); LPGAT1 and LIPC overexpression constructs were purchased from Genecopoeia (catalog #EX-mm15104-M61 and #EX-mm03119-M61). In brief, cells were transfected with 2 nm siRNA or DNA in serum-free cell culture media. INTERFERin reagent was added to the mix, incubated at room temperature for 10 min, and added to newly changed cell culture media for each well. Cells were incubated for 48–96 h before harvesting.

### Cellular fractionation

The NE-PER Nuclear and Cytoplasmic Extraction Reagent Kit was used to fractionate cells following manufacturer recommendations (catalog #78835, Thermo Fisher Scientific). In brief, extracellular fraction was collected by collecting the cell culture media from adherent cells. Adherent cells were washed using PBS and harvested by adding 2.5% trypsin in PBS (trypsin solution was filtered through a 0.22 μm filter before using; catalog #T9935 Sigma-Aldrich). Cells were centrifuged at 500 × *g* for 5 min and washed using PBS. The supernatant was removed, and the cell pellet was resuspended in ice-cold CER1 buffer using volumes recommended in the manufacturer protocol. The cells were vigorously vortexed for 15 s and incubated on ice for 10 min. Ice-cold CER2 buffer was added, vortexed for 5 s, and incubated on ice for 1 min. The sample was vortexed and centrifuged at 16,000 × *g* for 5 min. The supernatant (cytoplasmic fraction) was transferred to a clean tube. The pellet was resuspended in ice-cold NER buffer, vortexed, and incubated on ice for 40 min, with intermittent vortexing every 10 min. The sample was centrifuged at 16,000 × *g* for 10 min, and the supernatant (nuclear fraction) was transferred to a clean tube.

### Electrophoretic mobility shift assay

The LightShift Chemiluminescent EMSA Kit (catalog #20148, Thermo Fisher Scientific) and the Chemiluminescent Nucleic Acid Detection Module Kit (catalog #89880, Thermo Fisher Scientific) were used as instructed by the manufacturer recommendations. In brief, a precast DNA retardation gel (catalog #EC6365BOX, Thermo Fisher Scientific) was equilibrated, and the wells were flushed using 0.5× TBE buffer (5× TBE Buffer contained 450 mm Tris-Base, 10 mm EDTA, and 450 mm boric acid at pH 8.3). The gel was run without any samples for 30–60 min at 100 V. The binding reaction was prepared using the nuclear fraction from the cellular fractionation section: 0.5 μg of nuclear protein extract was incubated with 4 pmol biotin-labeled target DNA (biotin-5′-GATAAGTAGGGGAAAGGTCA-3′) in binding buffer (1× binding buffer, 2.5% glycerol, 5 mm MgCl2, 50 ng/μl poly deoxyinosinic-deoxycytidylic (dI-dC), 0.05% NP-40) for a total volume of 20 μl. The binding reaction was incubated at room temperature for 20 min. The samples were then resuspended in loading buffer (supplied in the kit) and run in an equilibrated precast DNA retardation gel for 60 min at 100 V. The membrane was activated, and samples were transferred as described in the Gel electrophoresis and immunoblotting section above. Membrane was UV cross-linked using the Stratalinker UV Crosslinker 1800 (Stratagene) using the “auto cross-link” function (60 s, 1200 μJ/cm^2^ × 100). Blocking buffer and wash buffer were prewarmed to 37°C. Membrane was blocked for 15 min with agitation followed by 15 min incubation with stabilized streptavidin-horseradish peroxidase conjugate in blocking buffer. Membrane was washed four times for 5 min each using wash buffer and equilibrated for 5 min using the substrate equilibration buffer, and while maintained it was protected from light. Membrane was incubated with the substrate working solution [luminol/enhancer solution + stable peroxide solution at a 1:1 (v/v) ratio] for 5 min and imaged using the ImageQuant LAS 4000 (catalog #08855-1327, GE Healthcare).

### Liposome extrusion and liposome flotation assay

Lipids were purchased at Avanti Polar Lipids (LPC 18:1; catalog #855773; and LPC 18:0; catalog #855774). Lipids were resuspended in chloroform, and 30 μg of lipid was aliquoted in glass vials, desiccated in speed vacuum, and resuspended in 100 μl of PBS using sonication. Lipids were extruded using the NanoSizer MINI Extruder Kit (catalog #TT-030–0001, T&T Scientific) through a 100 nm NanoSizer (catalog #TT-002–0010, T&T Scientific). Extruded liposomes (100 μl) were incubated with 2.5 μg (1 μl) of mouse optic nerve protein homogenate for 30 min at room temperature. Liposomes/micelles and protein mixture were then resuspended in 50% sucrose. The sucrose gradient was prepared by layering from bottom to top over 1 ml of liposome/micelle–protein mixture in 50% sucrose, 2 ml of 25% sucrose in PBS, and 1 ml of PBS. The gradient was ultracentrifuged at 114,000 × *g* at 4°C for 3.5 h. Fractions were collected using a mechanical pump to aspirate 1 ml of the bottom fraction followed by 2 ml of the middle fraction and 1 ml of the top fraction. The collected fractions were washed using PBS followed by centrifugation at 18,000 × *g* at 4°C for 25 min (rotor model GH-3.8, Beckman Coulter). The supernatant was discarded, and the pellet was processed for mass spectrometry, as described above.

### Protein–lipid overlay assay

The lipids listed above were dotted on a PVDF membrane at 30 μg/dot and allowed to completely dry. Cell culture lysate was depleted from endogenous lipids by adding 400 μl of a 1:1 (v/v) methanol/chloroform mix to 200 μl of cell culture lysate. Lysate was vortexed and incubated in ice for 5 min, followed by the addition of 350 μl of chloroform. Lysate was centrifuged at 18,000 × *g* for 15 min at 4°C. The organic layer containing lipids (bottom layer) was transferred to a new tube while the aqueous layer containing protein (top layer) was desiccated using the CentriVap Concentrator system. Protein was resuspended in 1× PBS. The membrane was incubated for 1 h at 4°C with 2.5 μg of cell culture lysate (OPCs, oligodendrocytes, and RGCs) in PBS, followed by cross-linking at 1200 × 100 μJ for 50 s (Stratalinker UV crosslinker; model 1800, Stratagene). The membrane was blocked, washed, and probed using primary and secondary antibodies, as described in the Gel electrophoresis and immunoblotting section above.

### Statistics

Data are expressed as the mean ± SE of three separate replicates. Statistical significance was assessed using paired (for PERG data) and unpaired (remaining data) *t* test with Prism software version 9 (GraphPad Software). *p* < 0.05 was considered statistically significant. Deimination levels were the measurement of raw densitometry for Cit-MBP normalized to MBP densitometry after membrane striping. Lipid abundance ratios were normalized to the protein concentration found in each sample used for lipidomic analysis. ANOVA was used for multiple-group comparisons using Prism software. MataboAnalyst version 4.0 was used to generate and analyze proteomics data. Reactome software version 75 was used to analyze protein pathways.

### Data availability

Mass spectrometry data (PRIDE archive accession: PXD024966, PXD025176, PXD025177, PXD025175; and Metabolomics Workbench archive accession: ST002047).

## Results

### LPC 18:1 is deficient in disease and has different biochemical behavior than LPC 18:0

**Figure 1.**
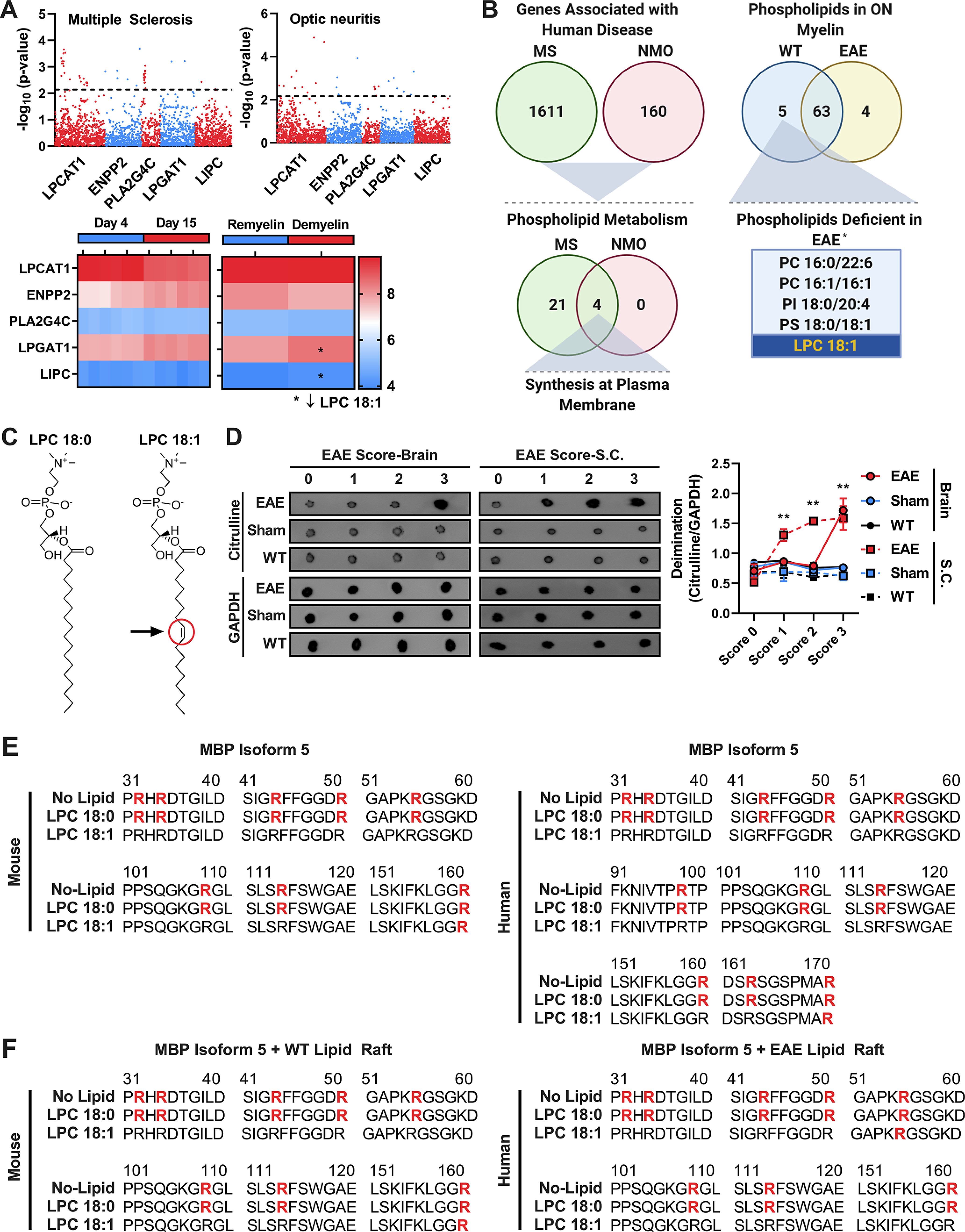
LPC 18:1 protects against hyperdeimination of MBP *in vitro*. ***A***, UK Biobank International Classification of Diseases PheWeb Analysis points toward genes associated with ocular manifestations of MS (UK BioBank: Multiple Sclerosis, accession number, 335; Optic Neuritis, accession number, 377.3). Gene expression analysis during different myelin conditions points toward elevated levels of LPGAT1 and LIPC in demyelinating conditions [GEO (Gene Expression Omnibus) accession number: GSE19403]. *Higher levels of these enzymes would metabolize LPC 18:1, resulting in lower levels of LPC 18:1. ***B***, Genes reported to have been associated in MS and NMO using the DisGeNET database [Multiple Sclerosis, concept unique identifier (CUI), C0026769; and Neuromyelits Optica, CUI C0027873]. Genes were curated for genes involved in phospholipid metabolism and subsequently compared for genes shared between MS and NMO. Shared genes were associated with plasma membrane synthesis. *Phospholipid profile comparison was taken from our previous study (Valdivia et al., 2020). ON, Optic nerve. Based on our previous study LPC 18:1 is the only lipid to protect against aberrant hyperdeimination. ***C***, Structural difference between LPC 18:0 and LPC 18:1. Arrow indicates the position of unsaturation in the acyl chain of LPC 18:1. ***D***, Dot blot analysis of levels of deimination in EAE brain and spinal cord (S.C.). Levels of deimination increase as the EAE clinical score increases (brain score 0, one-way ANOVA n.s.; brain score 1, one-way ANOVA n.s.; brain score 2, one-way ANOVA n.s.; brain score 3, one-way ANOVA, **p ≤ 0.01; S.C. score 0, one-way ANOVA n.s.; S.C. score 1, one-way ANOVA, **p ≤ 0.01; S.C. score 2, one-way ANOVA, **p ≤ 0.01; S.C. score 3, one-way ANOVA, **p ≤ 0.01). Citrulline, Millipore-MABS54887. *n* = 3. ***E***, Mass spectrometry analysis of mouse and human MBP isoform 5 deimination reaction in the presence of LPC 18:0 and LPC 18:1. Deiminated cites, Bold-red arginine (R). ***F***, Mass spectrometry analysis of mouse MBP isoform 5 deimination reaction in the presence of LPC 18:0 and LPC 18:1 and lipid rafts isolated from C57BL/6J WT or EAE mouse brain. Deiminated cites, Bold-red arginine (R). See Extended Data Figure 1-1.

Phenome-wide association study (PheWAS) analysis of MS and optic neuritis highlighted level alteration for five lipid metabolism genes (Fig. 1*A*, top). Gene expression during developmental myelination (control vs demyelination) identified two common genes with elevated expression levels associated with demyelination (Fig. 1*A*, bottom) that are consistent with the alteration of the CNS lyso-lipid pool. On the other hand, MS- and neuromyelitis optica (NMO)-associated gene changes also may result in predictive changes in phospholipid metabolism (Fig. 1*B*). These predictive changes converged with the lyso-lipid LPC 18:1 as a lipid deficient in the optic nerve in animal models of MS (Valdivia et al., 2020). LPCs are not bona fide components of the membrane bilayer, but they potentially serve diverse biological functions such as intracellular signaling (Cebecauer et al., 2018). LPCAT1, ENPP2, PLA2G4C, LPGAT1, and LIPC are enzymes that may act on both LPC 18:0 and LPC 18:1 and undergo a significant change during demyelination (Fig. 1*A*). The *K*_m_ values of ENPP2 and LPCAT1 are higher at V_max_ (maximum velocity) for LPC 18:1 than LPC 18:0, despite differing in a single bond in their chemical structure (Fig. 1*C*). LPGAT1 and LIPC are the enzymes for which LPC 18:1 (but not LPC 18:0; summarized in the BRENDA database) is the substrate. The higher levels of these two enzymes are consistent with a decrease in LPC 18:1 in demyelinating conditions (Dugi et al., 1995; Hill et al., 1996; McCoy et al., 2002; Yang et al., 2004; Yang and Subbaiah, 2015). LPC 18:1 is found deficient in both human MS patients and mouse model systems (Del Boccio et al., 2011; Valdivia et al., 2020). Consistent with prior findings in humans, the peptide-induced experimental autoimmune encephalomyelitis (EAE) mouse model of MS has an increase in total protein deimination that is proportionate to disease severity (Fig. 1*D*). Based on our elaborate experiments, we now report *in vitro* conservation of LPC 18:1-mediated protection against hyperdeimination of MBP5 from mouse to human (Fig. 1*E*,*F*; Valdivia et al., 2020), corroborating lipid complexation conferring protection against aberrant deimination.

10.1523/ENEURO.0429-21.2022.f1-1 Figure 1-1 Lipid raft isolation from mouse spinal cord and brain tissue. ***A***, EAE clinical score graph demonstrating the progression of clinical symptoms. LPC 18:1 injections were delivered to EAE animals when they reached a score of 2. ***B***, Immunoprecipitation of MBP from BL21 cells. MBP: catalog #ab7349, Abcam. MBP isoform 5 from both mouse and human were isolated. P, Pellet; W, wash discard; E, eluent. ***C***, Gradient setup for isolation of lipid rafts using sucrose density centrifugation. Sucrose fractions were collected in 18 fractions, but only 1–15 were used as fractions 16–18 are not typically considered raft fractions. ***D***, Western blot analysis of sucrose fractions 1–15 demonstrates that only fractions 1 and 2 of brain tissue are (+) for Flot1 and (–) for TfR. *This criterion is typical for lipid raft identification. ***E***, Total protein quantification and total cholesterol quantification for lipid raft fraction. Lipid rafts typically have low protein content and high cholesterol content, supporting the presence of lipid rafts in sucrose fractions 1 and 2. Download Figure 1-1, TIF file.

Our further investigations have corroborated the differential biochemical behavior of LPC 18:1 and LPC 18:0. This striking difference is interesting since LPC 18:0 only differs from LPC 18:1 by a single bond and LPC 18:0 is used to generate the lysolecithin mouse model of demyelination (Fig. 1*C*). The differential effect of LPC 18:0 and LPC 18:1 was evaluated in the EAE mouse model, using recombinant human and murine MBP5 (MS/remyelination-associated isoform; Fig. 1*F*, Extended Data Fig. 1-1*A*,*B*). Lipid rafts were isolated from controls and EAE via sucrose density centrifugation and were validated with markers (Extended Data Fig. 1-1*C–E*). Only MBP incubated with EAE (but not controls)-derived lipid rafts showed hyperdeimination, while EAE lipid rafts supplemented with LPC 18:1 protected MBP against hyperdeimination (Fig. 1*F*). LPC 18:1 protection of MBP hyperdeimination is consistent with our previous report, and this protection is conserved across mouse, bovine, human, and likely other mammalian species as well (Valdivia et al., 2020).

### LPC 18:1 optic nerve injections improve visual function in EAE mice

**Figure 2.**
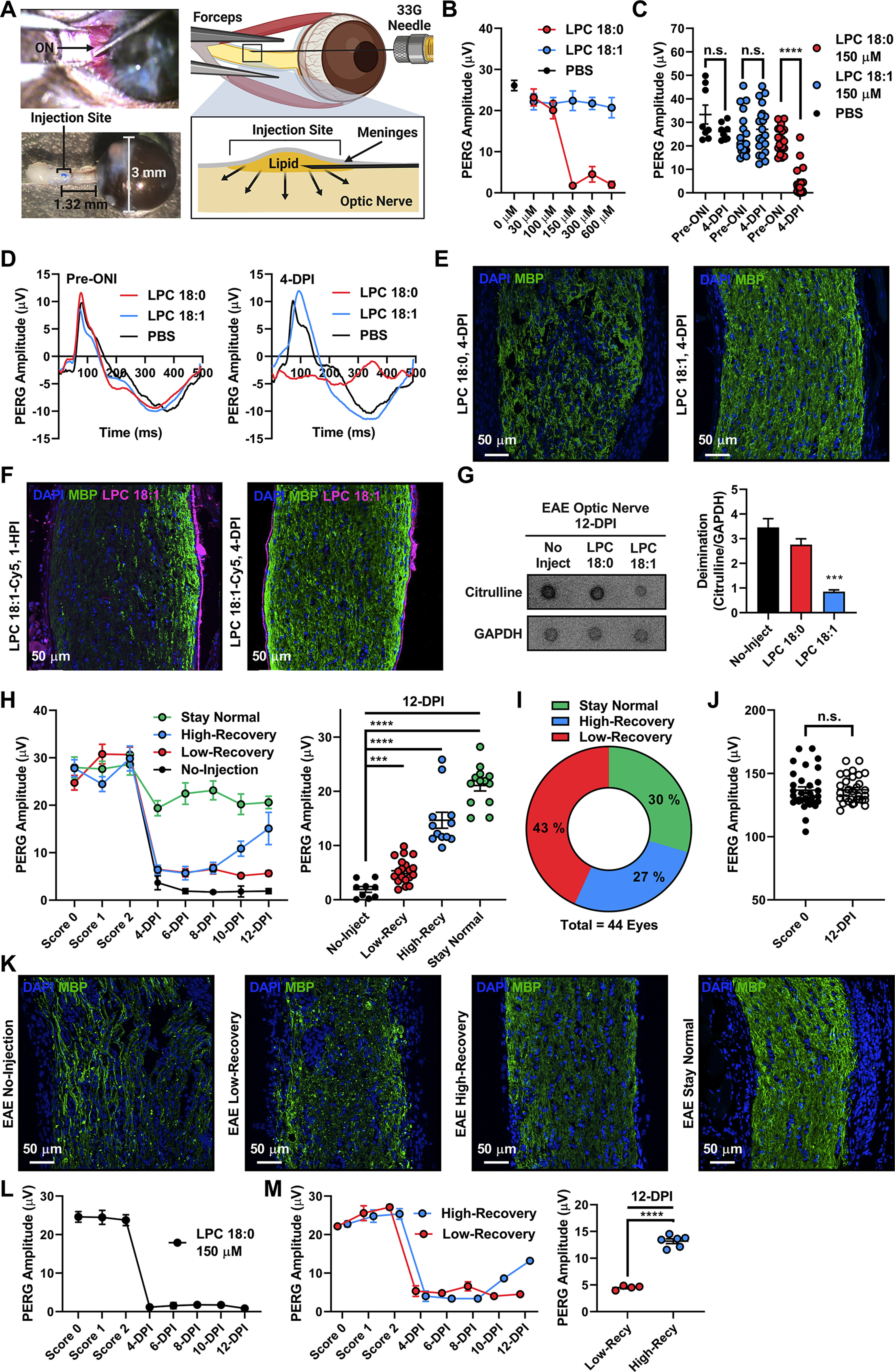
LPC 18:1 optic nerve injections improve visual function in EAE mice. ***A***, Surgical procedure for ON injections. Image created using BioRender. ***B***, Dose–response curve analysis for determining LPC 18:1 dose: 150 μm was the inflection point at which the control lipid LPC 18:0 decreased visual function while LPC 18:1 retained normal visual function. ***C***, Visual function analysis using PERG for optic nerve injections determined that 150 μm LPC 18:1 does not affect visual function (****p ≤ 0.0001; one-way ANOVA, ****p ≤ 0.0001). ***D***, PERG waves demonstrate that 150 μm optic nerve injection of LPC 18:1 does not affect visual function 4 days-post-injection (DPI). ***E***, Immunohistochemistry of C57BL/6J WT animals injected with LPC 18:0 and LPC 18:1 at 150 μm. MBP, Abcam, ab7349. Blue, DAPI; green, MBP. ***F***, Immunohistochemistry of C57BL/6J WT animals injected with 150 μm Cy5-LPC 18:1 1 h postinjection (HPI) and 4 DPI. MBP: catalog #ab7349 Abcam. Blue, DAPI; green, MBP; pink, Cy5-LPC 18:1. ***G***, Dot blot analysis of levels of deimination in the C57BL/6J-MOG35-55 EAE optic nerves injected with either LPC 18:0 or LPC 18:1. Levels of deimination are attenuated when injected with LPC 18:1 (***p ≤ 0.001; one-way ANOVA, ***p ≤ 0.001). Citrulline: MABS54887, Millipore. ***H***, PERG analysis of LPC 18:1 optic nerve injections in the C57BL/6J-MOG35-55 EAE animals at a clinical score of 2. The duration of the PERG measurements stopped 12 d post-LPC 18:1 optic nerve injection (DPI) when tissue was harvested. PERG analysis at 12 DPI demonstrates a statistical significance among all of the groups (***p ≤ 0.001; ****p ≤ 0.0001; one-way ANOVA, ****p ≤ 0.0001). ***I***, Composition of animals in each of the groups. ***J***, Flash ERG analysis demonstrates that the loss in visual function is not because of changes in inner retinal activity (photoreceptors) n.s., No-significance. ***K***, Immunohistochemistry of EAE animals injected with LPC 18:1 at 150 μm demonstrates increased myelination in the treated groups. MBP, catalog #ab7349, Abcam. Blue, DAPI; green, MBP. ***L***, PERG analysis of LPC 18:0 optic nerve injections in the C57BL/6J-MOG35-55 EAE animals at a clinical score of 2. Duration of the PERG measurements stopped 12 d post-LPC 18:1 optic nerve injection (DPI) when tissue was harvested. ***M***, PERG analysis of LPC 18:1 optic nerve injections in the SJL/J- PLP139-151 EAE animals at a clinical score of 2. The duration of the measurements stopped 12 DPI when tissue was harvested. PERG analysis at the 12 DPI demonstrates a statistical significance between the two groups (****p ≤ 0.0001; one-way ANOVA, ****p ≤ 0.0001). See Extended Data Figures 2-1, 2-2, and 2-3.

Next, we aimed to test whether *in vivo* supplementation of LPC 18:1 in the optic nerve can improve optic nerve function. To test this, we developed a new suboptic nerve sheath injection approach in C57BL/6J mice for direct delivery of LPC 18:1 into the optic nerve (Fig. 2*A*). We determined the appropriate nontoxic LPC 18:1 dose response to be 150 μm (subinflection point), while LPC 18:0 showed toxicity and PBS did not show any effect (Fig. 2*B*). LPC 18:0 but not LPC 18:1 injection showed a decrease in optic nerve function (lower PERG amplitude), suggesting that neither LPC 18:1 nor the procedure damages the optic nerve (Fig. 2*C–E*). The observed decreased optic nerve function because of LPC 18:0 is consistent with its known demyelinating effects, which were further confirmed using histologic examination (Fig. 2*E*). LPC 18:1 injection lacked such demyelination (Fig. 2*E*, Extended Data Fig. 2-2*A*).

10.1523/ENEURO.0429-21.2022.f2-1 Figure 2-1 EAE induction and optic nerve injection timeline. Animals were injected intraperitoneally with pertussis toxin (catalog #181, Biological Laboratories) a day before and a day after immunization with MOG35-55 (catalog #12668-05, BioSynthesis) emulsified in complete Freund’s adjuvant. Visual function was assessed using PERG. Animals were monitored for the development of EAE clinical scores until they reached an EAE score of 2, followed by optic nerve injections of LPC 18:1 or LPC 18:0 or PBS. Animal visual function was monitored 4, 6, 8, 10, and 12 d post-optic nerve injection (DPI). Optic nerves were harvested the day after and processed for further analysis. Download Figure 2-1, TIF file.

10.1523/ENEURO.0429-21.2022.f2-2 Figure 2-2 Immunohistochemistry (IHC) of optic nerves in WT and EAE mice. ***A***, Optic nerve of C57BL/6J WT mice injected with either LPC 18:0 or LPC 18:1 at 150 μm. MBP: catalog #ab7349, Abcam. Blue, DAPI; green, MBP. ***B***, Optic nerve of C57BL/6J WT mice injected with LPC 18:1-Cy5 at 150 μm. Tissue was harvested 1 h post injection (HPI) and 4 DPI. MBP: catalog #ab7349, Abcam. Blue, DAPI; green, MBP; pink, Cy5-LPC 18:1. ***C***, Optic nerve of EAE mice injected with LPC 18:1 at 150 μm. Tissue was harvested 12 d post-LPC 18:1 injection (DPI). MBP, catalog #ab7349 Abcam. Blue, DAPI; green, MBP. Download Figure 2-2, TIF file.

**Figure 3.**
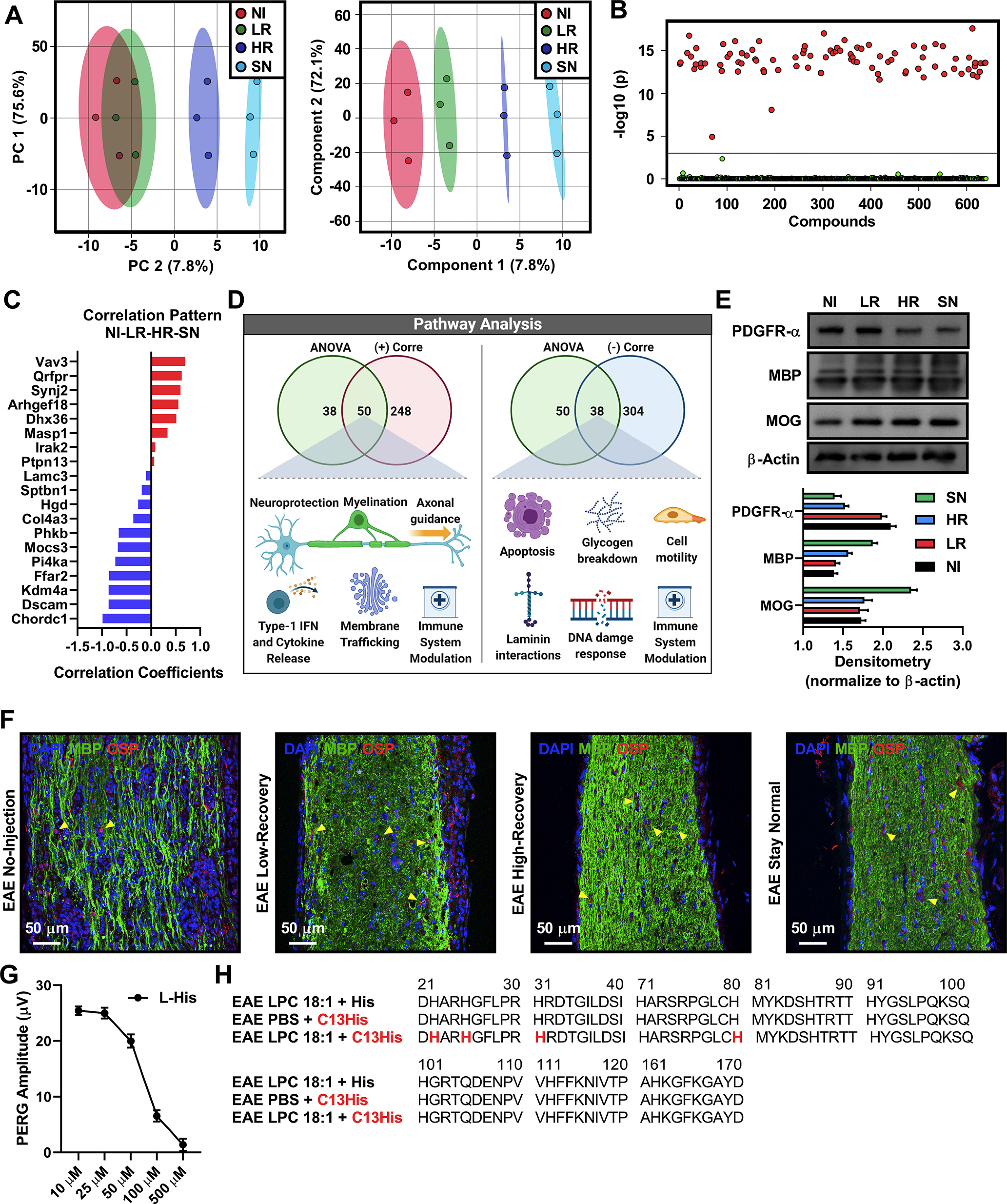
LPC 18:1 optic nerve injections promote remyelination in EAE mice. ***A***, Multivariable dimension reduction analysis (principal component analysis, left panel) and bilinear factor model analysis (partial least-squares discriminant analysis, right panel) demonstrating the separation of samples into four distinct groups. NI, No-injection control; LR, low-recovery group; HR, high-recovery group; SN, stay-normal group. ***B***, Quantitative iTRAQ mass spectrometry analysis of EAE optic nerves injected with LPC 18:1 (no-injection control, low-recovery, high-recovery, and stay-normal groups). One-way ANOVA demonstrating 88 significant proteins. Adjusted *p*-value cutoff = 0.001; *post hoc* analysis with Fisher’s LSD. ***C***, Correlation pattern analysis demonstrating the top 10 proteins that either increase (red) or decrease (blue) starting with the no-injection control group and progressively moving toward the SN group. Distance measure: Pearson R. ***D***, Reactome pathway analysis of proteins that are shared between the ANOVA and correlation pattern analysis (50 proteins for positive correlation and 38 proteins for negative correlation). Reactome pathways interactors were excluded, pathway *p*-value ≤ 0.05. ***E***, Western blot analysis demonstrating a negative correlation between oligodendrocyte progenitor cell markers and mature oligodendrocyte markers (PDGFR-α: one-way ANOVA, ***p ≤ 0.001; MBP: one-way ANOVA, ***p ≤ 0.001; MOG: one-way ANOVA, ***p ≤ 0.001). PDGFR-α, catalog #ab134123, Abcam; MBP: catalog #ab7349 Abcam; MOG, catalog #ab28766, Abcam. *n* = 3. NI, No-injection control group; LR, low-recovery group; HR, high-recovery group; SN, stay-normal group. ***F***, Immunohistochemistry of EAE animals injected with LPC 18:1 at 150 μm demonstrating mature oligodendrocyte localization (yellow arrowhead). MBP: catalog #ab7349, Abcam; OSP: catalog #ab7474, Abcam. Blue, DAPI; green, MBP; red, OSP. ***G***, Dose curve analysis for determining histidine dose: 10 μm was the concentration used for C13-histidine + LPC 18:1 EAE optic nerve injections. ***H***, Mass spectrometry C13-histidine + LPC 18:1 EAE optic nerve injections demonstrating the incorporation of isobaric C13-histidine in myelin basic protein. See Extended Data Figures 3-1, 3-2, 3-3, 3-4, and 3-5.

10.1523/ENEURO.0429-21.2022.f2-3 Figure 2-3 Electron microscopy of EAE optic nerves injected with LPC 18:1. Left, Representative coronal cross section for WT optic nerves (noninjected). Middle, Representative optic nerve for EAE mice in the high-recovery group (injected with LPC 18:1). Right, Representative optic nerve for EAE mice in the no-injection group. Yellow arrow points towards myelin sheath (dark rings). Myelin is observed to be compacted in the left and middle panels, whereas its less compact in the right panel. Download Figure 2-3, TIF file.

To determine the localization of LPC 18:1, fluorescent LPC 18:1-Cy5 was injected into the optic nerve of a C57BL/6J mouse. Following immediate optic nerve injections (1 h postinjection), LPC 18:1-Cy5 was distributed to the periphery of the optic nerve and continued to show persistence for 4 d (Fig. 2*F*, Extended Data Fig. 2-2*B*). This pattern may not be reflective of the true localization of LPC 18:1, as the large fluorescent tag attached to the head group can contribute to this pattern and cannot be completely ruled out; however, our imaging mass spectrometry (IMS) analyses are consistent with nontagged LPC 18:1 presenting the same pattern.

Next, LPC 18:1 was delivered to the optic nerve using the same approach (Fig. 2*A*) in EAE mice with a clinical score of 2 (Extended Data Figs. 1-1*A*, 2-1). Optic nerve deimination levels assessed 12 d postinjection (DPI) showed a decrease when injected with LPC 18:1, but not with LPC 18:0 or in the no-injection control (Fig. 2*G*). Clinical disability scores of 2 and 3 in EAE mice show a mild and severe decrease in optic nerve function, respectively; thus, a score of 2 enables a better readout of the LPC 18:1 protective effect than that of a score of 3, and hence they were selected for deimination analysis. EAE eyes demonstrated the following three distinct types of optic nerve responses to LPC 18:1 delivery: (1) optic nerve function was preserved at around baseline levels (stay normal); (2) a severe decrease in optic nerve function followed by a period of increased optic nerve function (High-Recovery); and (3) a drastic decrease in optic nerve function that was sustained throughout the study (Low-Recovery; Fig. 2*H*,*I*). The differential response to LPC 18:1 might be partly because of the EAE intrinsic pathology and cannot be completely ruled out. Our 12 DPI LPC 18:1 treatment demonstrated an increase in optic nerve function for all treatment groups compared with the no-injection control group (Fig. 2*H*). The majority of the EAE animals had a score of 3 at the 12 DPI time point, and LPC 18:1 showed a maximal vision protective restorative effect on all treated animals at this assessment point.

EAE mice were also assessed for outer retinal or photoreceptor activity to ensure that their decreased optic nerve function was not because of their inability to perceive light or photoreception (Fig. 2*J*). Further histologic and electron microscopy analysis showed consistency of the structural optic nerve health in the treated groups and demyelination in the EAE control group (Fig. 2*K*, Extended Data Figs. 2-2*C*, 2-3). The noninjected group, as expected, showed optic nerve demyelination, which gradually decreased in the LPC 18:1-injected group and positively correlated with improved optic nerve function (Fig. 2*H*,*I*). EAE mice injected with LPC 18:0, consistent with prior findings (Gregg et al., 2007), showed a sustained decrease in optic nerve function (Fig. 2*L*). Improvement in optic nerve function mediated by LPC 18:1 was also observed in the SJL/J- PLP_139-151_ EAE model (Fig. 2*M*), suggesting that the dramatic improvement in vision exerted *in vivo* by LPC 18:1 occurs in multiple demyelination models, regardless of genetic background.

Manifestations of optic neuritis in human subjects have been found to be heterogeneous, with an individual-intrinsic component (Klistorner et al., 2007). In the EAE model, similar intrinsic heterogeneity prevails, which contributes to heterogeneous manifestation in the optic nerve. However, pattern electroretinogram is a noninvasive technique that allows the objective measurement of optic nerve function in individual eyes (left and right). Therefore, the separate assessment of eyes that exhibit optic nerve demyelination can be identified in real time by the decrease in PERG amplitude. Noninjected EAE eyes that exhibited optic nerve demyelination (Fig. 2*K*) consistently demonstrated a decrease in PERG amplitude (Fig. 2*H*), while the ones that did not exhibited optic nerve demyelination remained within baseline levels (data not shown). As the EAE model exhibits heterogeneity in optic nerve demyelination, EAE animals were injected in both eyes to account for this heterogeneity. Therefore, EAE eyes in the stay-normal group reflect this heterogeneity, a condition in which these eyes might not have developed optic nerve demyelination or responded earlier to the effects of LPC 18:1 (Fig. 2*H*,*K*). On the other hand, EAE eyes that had a decrease in PERG amplitude postinjection reflect the subset of eyes that developed optic nerve demyelination (Fig. 2*H*,*K*, EAE Low-Recovery and High-Recovery). To address this heterogeneity, a more aggressive form of the EAE model was used (SJL/J- PLP_139-151_ EAE model). This aggressive model consistently developed optic nerve functional deficits in both eyes and responded similarly to LPC 18:1 injection (Fig. 2*L*,*M*). Despite heterogeneity and genetic background, the low-recovery and high-recovery EAE groups in both models demonstrated a substantial improvement in optic nerve function because of LPC 18:1 when compared with the no-injection group, attesting to the improving effects of this specific lyso-lipid (Fig. 2*H–M*).

### LPC 18:1 optic nerve injections promote remyelination in EAE mice

Next, we asked whether LPC 18:1 treatment-responsive vision recovery is because of the prevention of demyelination or because of the promotion of remyelination. Our labeled proteomics (iTRAQ) revealed that these four groups (low-recovery, high-recovery, stay-normal, and control no-injection groups) were compositionally distinct from each other (Fig. 3*A*). Proteins that were found statistically significant both in levels (Fig. 3*B*) and in the correlation pattern (Fig. 3*C*) were further analyzed for their role in biological pathways. Proteins that demonstrated a gradual increase in their correlation pattern were associated with processes such as myelination, axonal guidance, neuroprotection, and modulation of the immune system, whereas proteins with a gradual decrease were associated with DNA damage response, modulation of the immune system, and cell motility (Fig. 3*D*, Extended Data Fig. 3-3). These results suggested that LPC 18:1 might be promoting remyelination.

10.1523/ENEURO.0429-21.2022.f3-1 Figure 3-1 Isolation of myelin proteins from EAE optic nerves injected with isobaric C13-histidine. ***A***, Schematic experimental setup for isobaric optic nerve injections. Only newly synthesized protein should incorporate isobaric C13-histidine in its sequence. ***B***, Sucrose gradient ultracentrifugation of optic nerves (one optic nerve per tube). Myelin fraction is localized to the top of the gradient. ***C***, Validation of the presence of myelin proteins in myelin fraction of the sucrose gradient ultracentrifugation. MBP: catalog #ab7349, Abcam; MOG: catalog #ab28766, Abcam. ***D***, MBP enrichment for mass spectrometry analysis. MBP: catalog #ab7349, Abcam. ***E***, Mass spectrometry spectra for demonstrating incorporation of C13-histidine into MBP peptide sequence. Download Figure 3-1, TIF file.

10.1523/ENEURO.0429-21.2022.f3-2 Figure 3-2 LPC 18:1 optic nerve injections promote myelination and neuroprotection. Proteins identified using the liposome flotation assay complex directly with micelles made from LPC 18:1. Proteins labeled during the isobaric C13-histidine experiment are proteins that are newly synthesized as a consequence of LPC 18:1 signaling. iTRAQ analysis identifies proteins that have a gradual increase in abundance in the following pattern: No-Injection → Low-Recovery → High-Recovery → Stay-Normal. Cell-specific protein expression was referenced using the EMBL-EBI Expression Atlas database. Reactome pathway analysis demonstrated protective and beneficial pathways being activated. Download Figure 3-2, TIF file.

10.1523/ENEURO.0429-21.2022.f3-3 Figure 3-3 iTRAQ NI-LR-HR-SN. Download Figure 3-3, DOC file.

**Figure 4.**
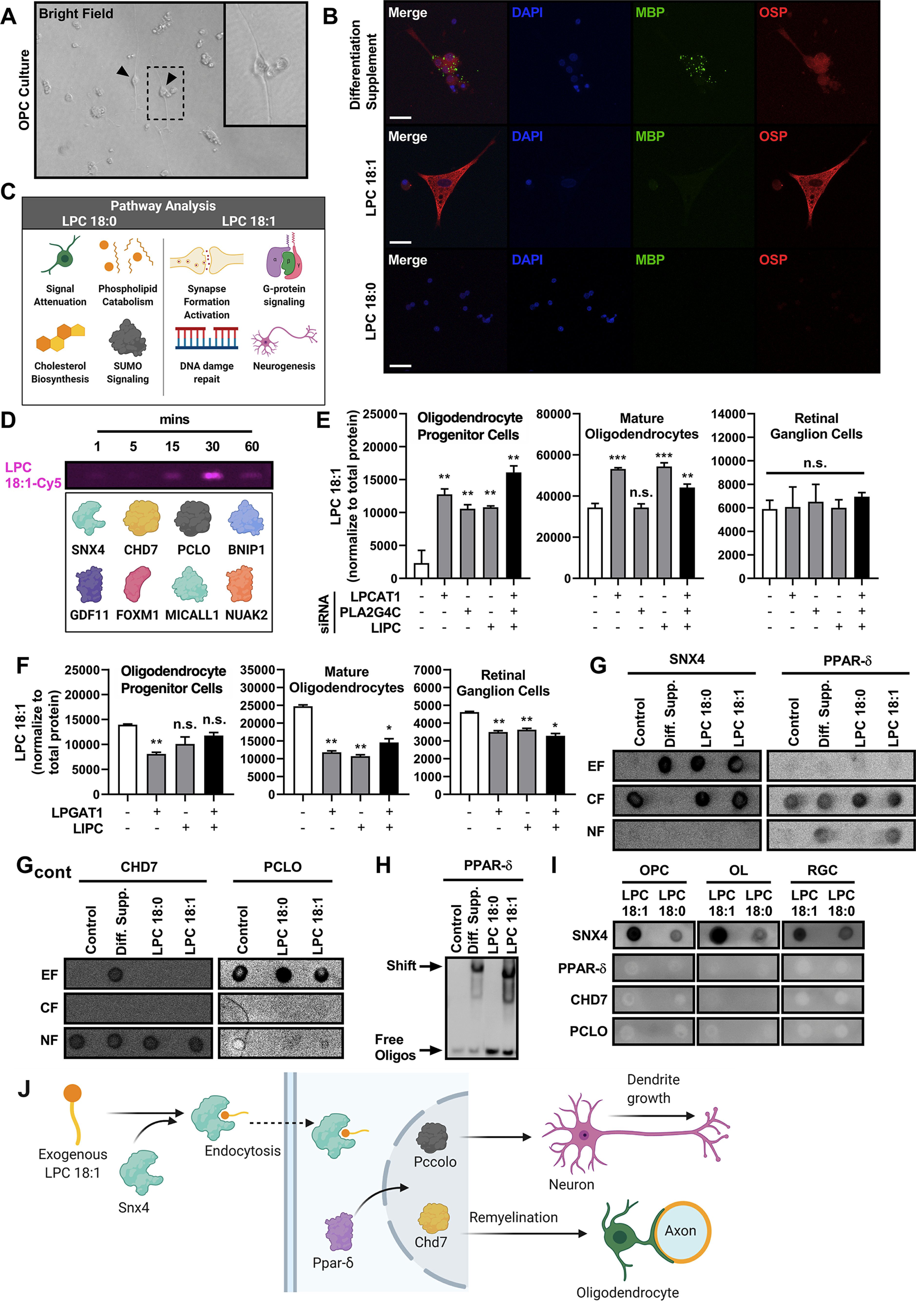
Oligodendrocyte maturation is mediated by LPC 18:1. ***A***, Rat oligodendrocyte progenitor cells culture depicts with typical OPC morphology (bright field). Arrows point toward OPCs, inlet panel represents the area in dotted square at a higher magnification. ***B***, Immunocytochemistry of rat OPCs treated with LPC 18:1 or LPC 18:0 at 10 μm, and C13-Histidine at 10 μm demonstrating LPC 18:1-mediated oligodendrocyte maturation. MBP: catalog #ab7349, Abcam; OSP: catalog #ab7474, Abcam. Blue, DAPI; green, MBP; red, OSP. Scale bar, 25 μm. ***C***, Reactome pathway analysis of proteins labeled with C13-histidine in the LPC 18:1 and LPC 18:0 OPC-treated groups. Reactome pathway interactors were excluded, pathway *p*-value ≤ 0.05. ***D***, OPC groups incubated with Cy5-LPC 18:1 at different time points followed by UV cross-linking. PhastGel identification of proteins crossed linked with Cy5-LPC 18:1 (pink band). ***E***, Relative abundance of LPC 18:1 in rat OPC, rat oligodendrocyte and mouse retinal ganglion cell cultures transfected with siRNAs. LPCAT1: catalog #s102346, Ambion; PLA2G4C: catalog #s107315, Ambion; LIPC: catalog #s67780, Ambion. ±SEM; *p ≤ 0.05, **p ≤ 0.01. ***F***, Relative abundance of LPC 18:1 rat OPC, rat oligodendrocyte, and mouse retinal ganglion cell cultures transfected with overexpression constructs. LPGAT1: catalog #Ex-mm15104-M61, Genecopoeia; LIPC: catalog #Ex-mm03119-M61, Genecopoeia. ±SEM; *p ≤ 0.05, **p ≤ 0.01. ***G***, OPC cell cultures treated with exogenous LPC 18:1 or LPC 18:0 followed by cellular fractionation. SNX4: catalog #ab198504, Abcam; PPAR-δ: catalog #ab23673, Abcam; CHD7: catalog #6505S, Cell Signaling Technology; PCLO: catalog #ab20664, Abcam. EF, Extracellular fraction; CF, cytosolic fraction; NF, nuclear fraction. ***H***, Nuclear fraction electrophoretic mobility shift assay (EMSA) of OPC cell cultures treated with exogenous LPC 18:1 or LPC 18:0. ***I***, protein–lipid overlay assay demonstrating the binding of selected proteins with LPC 18:1 and LPC 18:0. SNX4: catalog #ab198504, Abcam; PPAR-δ: catalog #ab23673, Abcam; CHD7: catalog #6505S, Cell Signaling Technology; PCLO: catalog #ab20664, Abcam. ***J***, Proposed model for LPC 18:1-mediated remyelination through oligodendrocyte maturation. See Extended Data Figures 4-1, 4-2, 4-3, 4-4, 4-5, and 4-6.

10.1523/ENEURO.0429-21.2022.f3-4 Figure 3-4 Isobaric C13-histidine. Download Figure 3-4, DOC file.

10.1523/ENEURO.0429-21.2022.f3-5 Figure 3-5 Proteins complexing with LPC 18:1 and LPC 18:0 micelles. Download Figure 3-5, DOC file.

To determine how the optic nerve myelination pattern might be related to known myelination biology, Western blot and immunohistochemistry analyses were conducted. We found that LPC 18:1-treated groups inversely correlated with oligodendrocyte progenitor cells (PDGFR-α) markers and positively correlated with mature oligodendrocyte (MBP, MOG) markers (Fig. 3*E*). We further found that in LPC 18:1-treated optic nerves, mature oligodendrocytes localize to areas of myelination (Fig. 3*F*). These findings suggested that LPC 18:1 promotes remyelination through oligodendrocyte maturation. We also demonstrated that LPC 18:1 acts via MBP to maintain deimination patterns in EAE mice, which we have previously reported to promote structural stability of the myelin sheath (Figs. 1*E*,*F*, 2*G*). This suggests the notion that remyelination might be sustained by a secondary effect in maintaining deimination patterns of myelin proteins.

To further test this hypothesis, another cohort of EAE mice was injected with a mixture of LPC 18:1 + isobaric C13-histidine. We standardized and validated this new assay, wherein incorporation of C13-histidine in bona fide myelin proteins would suggest remyelination (Extended Data Fig. 3-1*A*). We found that 10 μm isobaric C13-histidine was the optimal concentration (Fig. 3*G*). Isobaric C13-histidine was found to be incorporated into MBP only from the LPC 18:1 group, suggesting new MBP5 synthesis (Fig. 3*H*, Extended Data Fig. 3-1*B–E*). Pathway analysis of C13-histidine-labeled proteins showed an association with myelination, axonal guidance, and receptor/channel activation, supporting the notion of oligodendrocyte maturation and remyelination (Extended Data Figs. 3-2, 3-4). Receptor/channel activation was the pathway that suggested LPC 18:1-exerted effects might be receptor mediated; therefore, a liposome flotation assay was conducted using LPC 18:1 and LPC 18:0 micelles to identify proteins that are directly complexing with LPCs. Four proteins (TLR1, ANO1, NKD1, and NOM1) were identified to differentially complex with LPC 18:1, offering insight into the potential mechanism of action (Extended Data Figs. 3-2, 3-5). This highlights the diverse biological effects of LPC 18:1, which include an initial potential to promote oligodendrocyte maturation (Fig. 3*E*), followed by the promotion of remyelination (Fig. 3*F*,*H*) and the maintenance of myelination by maintaining homeostatic deimination patterns in myelin proteins (Figs. 1*E*,*F*, 2*G*).

### LPC 18:1 mediates oligodendrocyte maturation

To understand the effects of LPC 18:1 at the isolated cellular level, rat OPCs were cultured and incubated in the presence of LPC 18:1 and LPC 18:0 (Fig. 4*A*, Extended Data Fig. 4-1). LPC 18:1-exposed (24 h postincubation) OPCs showed differentiation into mature oligodendrocytes compared with controls. LPC 18:0 lacked this maturation effect (Fig. 4*B*, Extended Data Fig. 4-2). OPC cultures incubated in the presence of isobaric C13-histidine with either LPC 18:1 or LPC 18:0 showed protein synthesis consistent with observed cellular-level effects (Fig. 4*C*). LPC 18:1-treated cells showed newly synthesized proteins for cell proliferation, cell maturation, and activation, G-protein signaling, while LPC 18:0-treated group showed pathways such as signal attenuation and phospholipid catabolism (Fig. 4*C*), which lends support that LPC 18:1 promotes oligodendrocyte maturation. To investigate the direct complexation of LPC 18:1 with OPC proteins, OPCs were incubated in the presence of LPC 18:1-Cy5 and UV cross-linked at different time points (1–60 min). Subsequent isoelectric focusing gel fractionation and mass spectrometry identified several proteins cross-linked to LPC 18:1-Cy5 (Fig. 4*D*). These included proteins known to be involved in oligodendrocyte maturation, such as SNX4 and CHD7 (Fig. 4*D*,*J*). PCLO is a protein identified as being associated with neurogenesis; therefore, a subset of experiments using mouse RGCs were performed.

10.1523/ENEURO.0429-21.2022.f4-1 Figure 4-1 LPC 18:1 oligodendrocyte progenitor cell differentiation experimental setup. Experimental setup for rat OPC cultures. OPC were plated at 3 × 10^4^ cells per well/chamber (10 μl for 96-well plate and 30 μl for slide chamber). OPCs were incubated at 37°C for 24 h (incubated >24 h if cells were not fully attached). Three different experimental setups included the following: (1) plating and exposing OPCs to LPC 18:1-Cy5 (10 μm) followed by UV cross-linking, cell lysis, and PhastGel separation; (2) plating and exposing OPCs to differentiation supplement as a positive control, LPC 18:0 (10 μm), LPC 18:1 (10 μm), and PAD followed by cell lysis and mass spectrometry analysis. Only the treated groups included isobaric C13-histidine; and (3) plating and exposing OPCs to LPC 18:0 (10 μm), LPC 18:1 (10 μm), and PAD followed by fixation using 4% paraformaldehyde (PFA) and imagining. Download Figure 4-1, TIF file.

10.1523/ENEURO.0429-21.2022.f4-2 Figure 4-2 Rat OPCs treated with LPC 18:1. ***A***, Immunohistochemistry of rat OPCs treated with LPC 18:1. MBP: catalog #ab7349, Abcam; OSP: catalog #ab7474, Abcam. Blue, DAPI; green, MBP; red, OSP. Scale bar, 25 μm. ***B***, Rat OPC cell culture treated with LPC 18:1 (bright field). Download Figure 4-2, TIF file.

10.1523/ENEURO.0429-21.2022.f4-3 Figure 4-3 Lyso-lipid metabolic enzymes mediate levels of LPC 18:1 experimental setup. ***A***, Experimental setup for silencing of LPC metabolic enzymes via siRNA. Rat OPCs and mouse RGCs were plated at 3 × 10^4^ cells/well/chamber (10 μl for 96-well plate and 30 μl for slide chamber). OPCs and RGCs were incubated at 37°C for 24 h (incubated >24 h if cells were not fully attached). OPCs were either differentiated into oligodendrocytes through the addition of differentiation supplement. OPCs, oligodendrocytes and RGCs were transfected with siRNAs for LPCAT1, PLA2G4C, and LIPC followed by cell lysis and mass spectrometry lipidomic analysis for LPC 18:1 levels. ***B***, The same experimental setup as described above was implemented with the exception of cell transfection. Cells were transfected with an overexpression construct for LPGAT1 and LIPC. Cells were lysed and analyzed by mass spectrometry for levels of LPC 18:1. Download Figure 4-3, TIF file.

10.1523/ENEURO.0429-21.2022.f4-4 Figure 4-4 Cellular fractionation and identification of proteins of interest experimental setup. Experimental setup for rat OPC cultures. OPCs were plated at 3 × 10^4^ cells/well (10 μl for 96-well plate). OPCs were incubated at 37°C for 24 h (incubated >24 h if cells were not fully attached) followed by treatment with differential supplement control, LPC 18:0 (10 μm) or LPC 18:1 (10 μm). Cells were incubated for 24 h followed by media collection (extracellular fraction) and NE-PER cellular fractionation. Cellular fractions were analyzed using Western blot for the presence of proteins of interest (SNX4, PPAR-δ, CHD7, and PCLO). The nuclear fraction was further analyzed via electrophoretic mobility shift assay. Download Figure 4-4, TIF file.

10.1523/ENEURO.0429-21.2022.f4-5 Figure 4-5 OPC cell culture with siRNA and overexpression construct. ***A***, Representative cell culture morphology for rat OPCs treated with siRNA, rat oligodendrocytes (OL) treated with siRNA and mouse RGCs treated with siRNA. ***B***, Representative cell culture morphology for rat OPCs treated with overexpression construct, rat oligodendrocytes (OLs) treated with overexpression construct and mouse RGCs treated with overexpression. OPC and OL cultures were validated using mRNA and protein analyses following published articles (Jäkel et al., 2019). Download Figure 4-5, TIF file.

10.1523/ENEURO.0429-21.2022.f4-6 Figure 4-6 Proposed model for LPC 18:1-mediated immune response. Top left model depicts the levels of protein (P) and deimination (D) during healthy conditions. Both levels are equal. Bottom left model depicts the levels of P and D during multiple sclerosis. Protein levels are stable; however, the levels of deimination increase. ***A***, Increase in deimination and the presence of LPC 18:1 permit the development of oligodendrocyte progenitor cells into mature oligodendrocytes. This process is permissible for myelination. ***B***, During myelination, MBP maintains baseline levels of deimination and a close conformation. This close conformation allows for the formation of a stable compact myelin sheath. ***C***, In the absence of LPC 18:1, oligodendrocyte progenitor cells cannot fully differentiate, which leads to an accumulation of aberrant hyperdeimination. Increased hyperdeimination on MBP is associated with priming of the immune system. ***D***, Aberrant hyperdeimination of MBP is also associated with an open conformation, which makes MBP more susceptible to proteolysis. This increase in proteolysis depletes MBP from the myelin sheath, making it unstable and susceptible to demyelination. All of these events contribute to the autoimmune response observed in multiple sclerosis. Download Figure 4-6, TIF file.

Together, these findings suggest SNX4-mediated transport of exogenous LPC 18:1 into the cytosol can induce nuclear translocation of proteins that can cause changes in gene expression such as the transcription factor PPAR-δ (Fig. 4*J*). We next used siRNA against enzymes known to metabolize LPCs (LPCAT1, PLA2G4C, and LIPC) on OPC and RGC cultures (Extended Data Figs. 4-3, 4-5*A*). We measured levels of LPC 18:1 in all experiments subjected to enzyme modulation. siRNA inhibition of enzymes (LPCAT1, PLA2G4C, and LIPC) showed an increased level of LPC 18:1 (Fig. 4*E*). Conversely, we overexpressed recombinant LPGAT1 and LIPC proteins in OPCs and RGCs, which resulted in decreased levels of LPC 18:1 (Fig. 4*F*, Extended Data Figs. 4-3, 4-5*B*). These results are consistent with modulation of the LPC 18:1 level mediated by these enzymes (Fig. 4*F*), which were differentially identified to undergo alteration in human MS and optic neuritis (Fig. 1*A*,*B*). Cultured OPCs and RGCs treated with exogenous LPC 18:1 showed SNX4 translocation into the cytoplasm while PPAR-δ, CHD7, and PCLO translocated to the nucleus, respectively (Fig. 4*G*, Extended Data Fig. 4-4). The active status of nuclear translocated PPAR-δ was confirmed by electrophoretic mobility shift assay, which demonstrated an upward shift (Fig. 4*H*, Extended Data Fig. 4-4).

Our studies indicate that LPC 18:1 exerts its effect to improve optic nerve function by promoting remyelination and partly by promoting neurogenesis and deimination homeostasis. We have also investigated the underlying mechanism of LPC 18:1 action. We identified the interaction of SNX4, a protein critical for endocytosis (Lingelem et al., 2020), with LPC 18:1 (Fig. 4*I*). SNX4-mediated endocytosis requires the recruitment of several proteins. The modulation of these interactions depends on membrane lipid composition (Lingelem et al., 2020) and involves a glycosylphosphatidylinositol-anchored proteins-enriched early endosomal compartment (Kumari et al., 2010; Lingelem et al., 2020). The presence of SNX4-mediated alkyl-lyso-phospholipid endocytosis, reported earlier, supports our findings (Cuesta-Marbán et al., 2013). LPC 18:1 mediated the activation of PPAR-δ in human skeletal muscle cells (Klingler et al., 2016), which is also consistent with our finding of activation of PPAR-δ in oligodendrocytes and RGCs (Fig. 4*G*,*H*). PPAR-δ is known to complex with RXR-α (retinoid X receptor-α) and in turn complex with CHD7, a protein also identified in our experiments (Fig. 4*D*,*G*). CHD7 has been shown to regulate the onset of remyelination in the CNS (He et al., 2016; Klingler et al., 2016). These studies suggest that LPC 18:1 is internalized within cells by endocytosis and activates signaling mediated by PPAR-δ, which in turn activates CHD7 in rat OPCs and PCLO in mouse RGCs promoting cell maturation, remyelination, and growth of RGCs, respectively.

## Discussion

In demyelinating diseases and in MS, the presence of hyperdeimination in the brain and other CNS tissue was recorded more than 3 decades ago (Whitaker et al., 1992). However, the occurrence of hyperdeimination during early development presented a paradox (Moscarello et al., 1994). Neuron-ectopic hyperdeimination in demyelinating diseases finally resolved this paradox. The serendipitous analysis of retina because of MS-associated vision loss was critical toward this resolution (Enriquez-Algeciras et al., 2013). In contrast to the brain, the relative lack of astroglial cells within the retina helped to recognize simultaneous hypodeimination in neurons and hyperdeimination in other cells. This contributed to recognition of neuron-ectopic hyperdeimination as a pathologic component during demyelination (Bhattacharya et al., 2008). Immature oligodendrocytes have long been known to undergo deimination and potentially aberrant hyperdeimination in MS (Akiyama et al., 1999). Recently, it was shown that deimination is necessary for oligodendrocyte differentiation and myelination (Falcao et al., 2019). With the recognition of processivity of PADs and PAD2 in particular, we conjectured protein–lipid complexation to protect against aberrant hyperdeimination and potentially to restore optic nerve function during demyelination (Valdivia et al., 2020).

Here we show conservation of LPC 18:1 protection of MBP5 from aberrant hyperdeimination across different mammalian species, which is consistent with a generalized mechanism in mammals. Deficiency of specific lipids such as LPC 18:1 (Fig. 1*A*,*B*) thus results in aberrant PAD access and deimination, leading to autolysis and initiation of an immunogenic cascade in demyelinating diseases (Musse et al., 2006). PheWAS genomic analyses of the developmental onset of myelination (control vs demyelination; Fig. 1*A*) showed differences in ENPP2, LPCAT1, PLA2G4C, LPGAT1, and LIPC enzymes during myelination. ENPP2, LPCAT1, and PLA2G4C may act on both LPC 18:0 and LPC 18:1 (as described by their enzymatic properties in the BRENDA database). However, changes in LPGAT1 and LIPC, which act only on LPC 18:1 but not LPC 18:0, alone can account for the observed deficiency or absence of LPC 18:1, thus suggesting the importance of lipid metabolism in demyelinating disease pathology.

Our comparison of phospholipid metabolism differences in MS and EAE provides insight into lipid deficiencies and the potential aberration of lipid-metabolizing enzymes, consistent with observed LPC 18:1 deficiency (Fig. 1*B*). LPC 18:1 as a conserved lipid protecting against aberrant hyperdeimination and restoring optic nerve function (Figs. 1, 2) is surprising, given that its structurally related homolog LPC 18:0 is a well established toxin-induced demyelinating agent. This is the second instance that a remarkable contrast in biological function is attributed to a change in a single bond (Chaurasia et al., 2019) and likely one of the very first for the CNS. Recent research has emphasized the role of lipids in regeneration and remyelination simultaneously (Uyeda and Muramatsu, 2020; Berghoff et al., 2021). Pharmacological remyelinating agents dramatically improve visual dysfunction, suggesting the possibility of an underlying strong neuronal regeneration component in addition to remyelination (Sekyi et al., 2021). Thus, it is unsurprising to observe similar effects mediated by LPC 18:1. Here, we found a link to PPAR-δ, CHD7, and PCLO activation in oligodendrocytes and neurons, respectively; however, the further detailed nature of this link remains to be uncovered.

Two paradigms for the pathogenesis of demyelinating diseases propose an initial event triggering an autoimmune response occurring outside the CNS (outside-in) or inside the CNS (inside-out; ‘t Hart et al., 2021). Consensus between these two paradigms has yet to be reached, with evidence supporting both hypotheses. Our proposed model for LPC 18:1 deficiency leading to aberrant PAD2-mediated hyperdeimination would explain the observed increase in immunogenic MBP peptides, supporting the notion of an inside-out paradigm (Extended Data Fig. 4-6). Our preclinical data uncovered a striking difference in biological behavior in the CNS mediated by a change in a single bond. LPC 18:1 was demonstrated to confer protection against protein hyperdeimination, shift the equilibrium of OPCs to mature oligodendrocytes, and promote 0restoration of optic nerve function, contrasting the toxic effects of LPC 18:0. These findings suggest a potential translational application for a spectrum of diseases with heterogenous optic nerve demyelination.

## Synthesis

Reviewing Editor: Erica Glasper, The Ohio State University Decisions are customarily a result of the Reviewing Editor and the peer reviewers coming together and discussing their recommendations until a consensus is reached. When revisions are invited, a fact-based synthesis statement explaining their decision and outlining what is needed to prepare a revision will be listed below. The following reviewer(s) agreed to reveal their identity: Ahdeah Pajoohesh-Ganji, Lu Sun. Note: If this manuscript was transferred from JNeurosci and a decision was made to accept the manuscript without peer review, a brief statement to this effect will instead be what is listed below.

Synthesis: Using several techniques, this manuscript provides evidence for the therapeutic use of LPC 18:1, but not LPC 18:0, in restoring optic nerve myelination via oligodendrocytes maturation. This findings from these experiments have translational promise for restoring optic nerve functioning following demyelinating diseases. The reviewers agree that this manuscript will advance the field of demyelination and/ or remyelination and add to our understanding of mechanisms promoting myelin repair.

Overall, the reviewers enthusiastically supported the manuscript, however, there are a few major and minor concerns that should be addressed. Where appropriate, reviewer’s specific comments were copied and pasted into this synthesis. They can be found in <i>italicized text</i>.

Major Concerns:

•The authors are encouraged to add more discussion concerning the lack of consistent demyelination throughout the CNS with this model of EAE. Interpretation of the findings regarding full optic nerve recovery should be reconsidered given the model’s lack of consistency.

•<i>Regarding the in vitro mechanistic studies in Figure 4, although the method section has documented the catalog number of the OPCs purchased, it’s better to specify that the OPCs here are rat OPCs. And in Figure 4B, the LPC 18:1 treated panel does not look like the stereotypical morphology of differentiating oligodendrocytes. Is it possible to provide a larger view of this culture instead of showing one single representative cell?</i>

Minor Concerns:

•The authors are encouraged to carefully reconsider their use of the term “immature oligodendrocytes”. <i>Immature oligodendrocyte or pre-myelinating oligodendrocyte (pre-OLs) is a designated cell stage during oligodendrocyte development, which expresses certain markers, such as ENPP6 and BCAS1</i>. Clarified terminology is requested as the authors may be referring to oligodendrocyte precursor cells (OPCs) but instead used the term pre-OLs. OPCs are not equivalent to immature oligodendrocytes. This clarification should be made within the main text of the manuscript as well as in the figure legends. For example, Figure 4E and 4F compare OPCs and mature oligodendrocytes, but the graphs are labeled as “immature oligodendrocytes”.

•The authors demonstrate via the proteomics analysis and the cellular studies that LPC 18:1 <i>improves visual function recovery by inducing OPC differentiation and promoting remyelination</i>, however, prior studies and data presented Figure 1 also revealed LPC 18:1 protects myelin basic protein against hyper-deimination, preventing additional demyelination. Therefore, it is suggested that the authors increase their discussion of the dual function of LPC 18:1 when discussing Figure 3 within the text.
