# Supplementary figures and images for: Lyso-Lipid-Induced Oligodendrocyte Maturation Underlies Restoration of Optic Nerve Function
Source: eNeuro. 2022 Jan 24;9(1):ENEURO.0429-21.2022. doi: 10.1523/ENEURO.0429-21.2022 (PMC8805197; doi:10.1523/ENEURO.0429-21.2022)

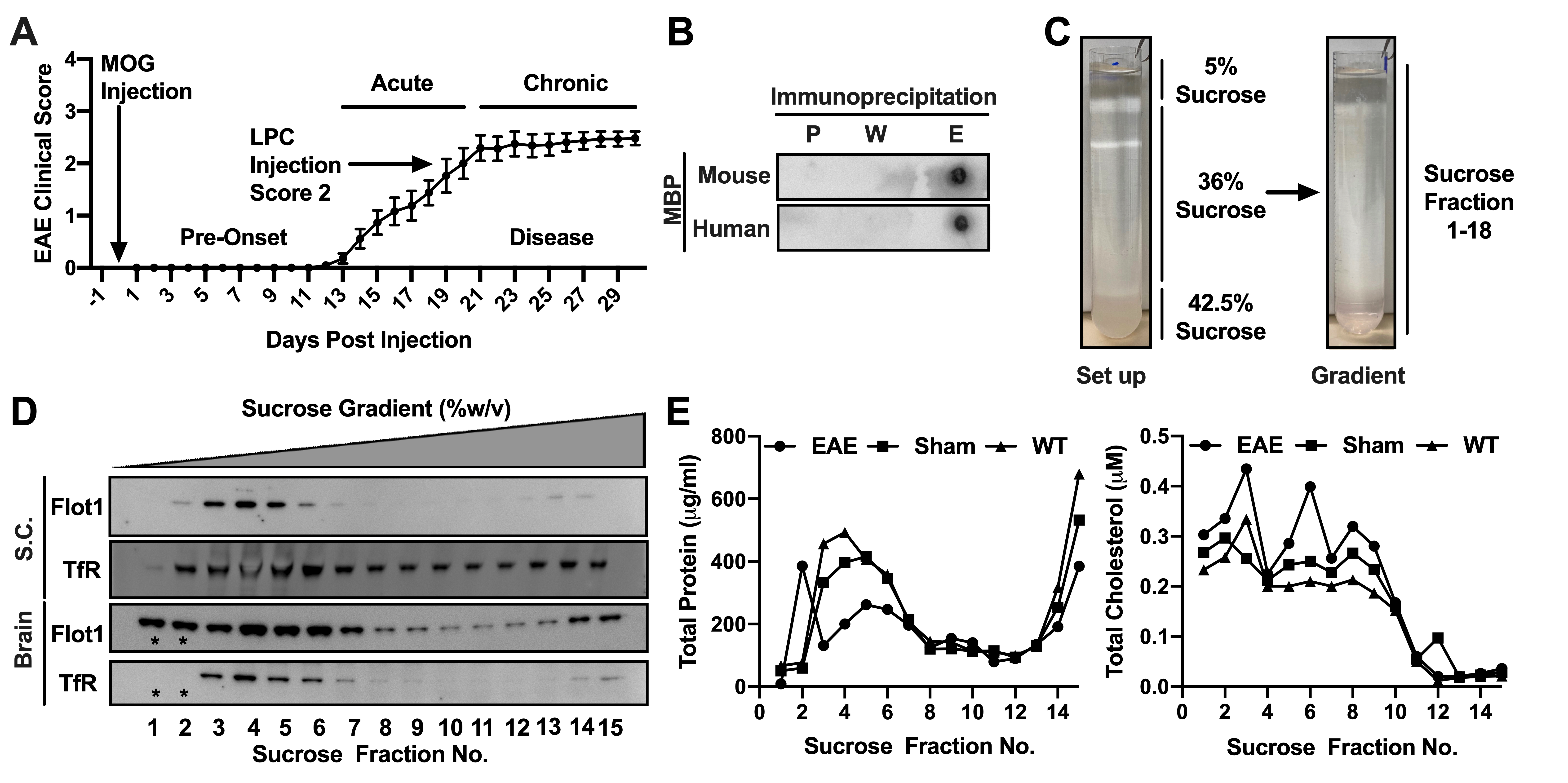

Supplement: Figure 1-1 — Lipid raft isolation from mouse spinal cord and brain tissue. A, EAE clinical score graph demonstrating the progression of clinical symptoms. LPC 18:1 injections were delivered to EAE animals when they reached a score of 2. B, Immunoprecipitation of MBP from BL21 cells. MBP: catalog #ab7349, Abcam. MBP isoform 5 from both mouse and human were isolated. P, Pellet; W, wash discard; E, eluent. C, Gradient setup for isolation of lipid rafts using sucrose density centrifugation. Sucrose fractions were collected in 18 fractions, but only 1–15 were used as fractions 16–18 are not typically considered raft fractions. D, Western blot analysis of sucrose fractions 1–15 demonstrates that only fractions 1 and 2 of brain tissue are (+) for Flot1 and (–) for TfR. *This criterion is typical for lipid raft identification. E, Total protein quantification and total cholesterol quantification for lipid raft fraction. Lipid rafts typically have low protein content and high cholesterol content, supporting the presence of lipid rafts in sucrose fractions 1 and 2. Download Figure 1-1, TIF file. [file enu-eN-NWR-0429-21-s01.tif]

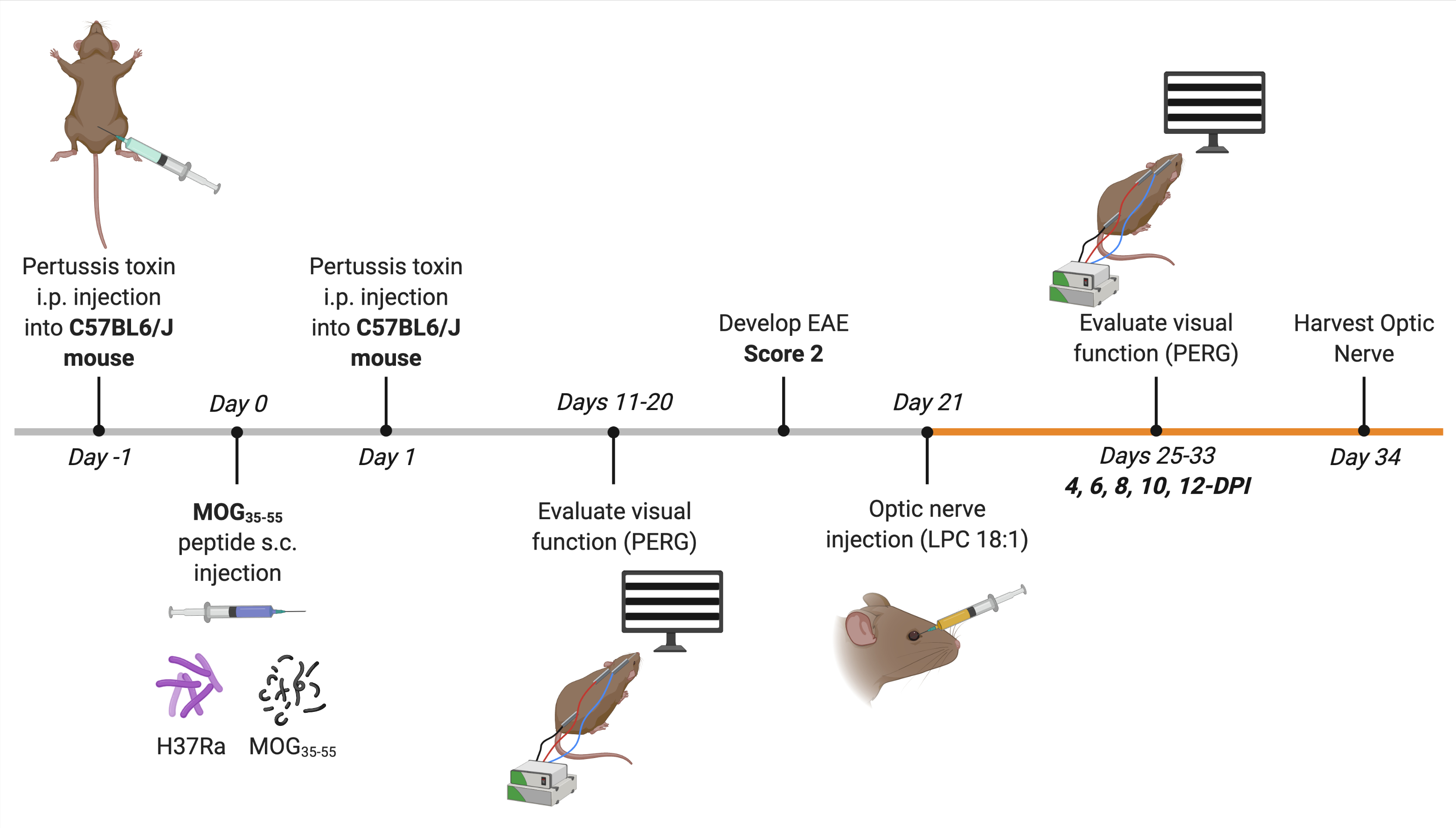

Supplement: Figure 2-1 — EAE induction and optic nerve injection timeline. Animals were injected intraperitoneally with pertussis toxin (catalog #181, Biological Laboratories) a day before and a day after immunization with MOG35-55 (catalog #12668-05, BioSynthesis) emulsified in complete Freund’s adjuvant. Visual function was assessed using PERG. Animals were monitored for the development of EAE clinical scores until they reached an EAE score of 2, followed by optic nerve injections of LPC 18:1 or LPC 18:0 or PBS. Animal visual function was monitored 4, 6, 8, 10, and 12 d post-optic nerve injection (DPI). Optic nerves were harvested the day after and processed for further analysis. Download Figure 2-1, TIF file. [file enu-eN-NWR-0429-21-s02.tif]

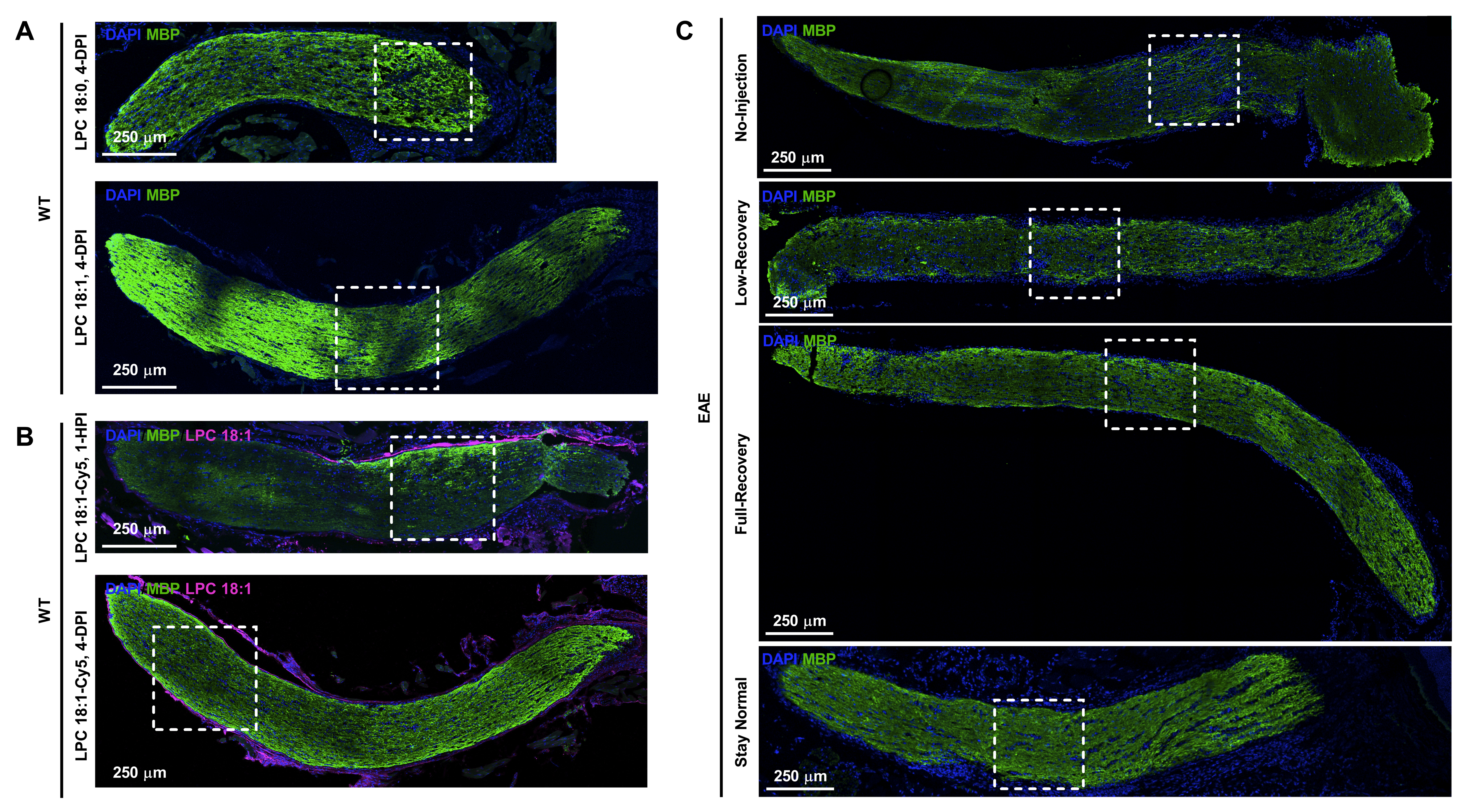

Supplement: Figure 2-2 — Immunohistochemistry (IHC) of optic nerves in WT and EAE mice. A, Optic nerve of C57BL/6J WT mice injected with either LPC 18:0 or LPC 18:1 at 150 μm. MBP: catalog #ab7349, Abcam. Blue, DAPI; green, MBP. B, Optic nerve of C57BL/6J WT mice injected with LPC 18:1-Cy5 at 150 μm. Tissue was harvested 1 h post injection (HPI) and 4 DPI. MBP: catalog #ab7349, Abcam. Blue, DAPI; green, MBP; pink, Cy5-LPC 18:1. C, Optic nerve of EAE mice injected with LPC 18:1 at 150 μm. Tissue was harvested 12 d post-LPC 18:1 injection (DPI). MBP, catalog #ab7349 Abcam. Blue, DAPI; green, MBP. Download Figure 2-2, TIF file. [file enu-eN-NWR-0429-21-s03.tif]

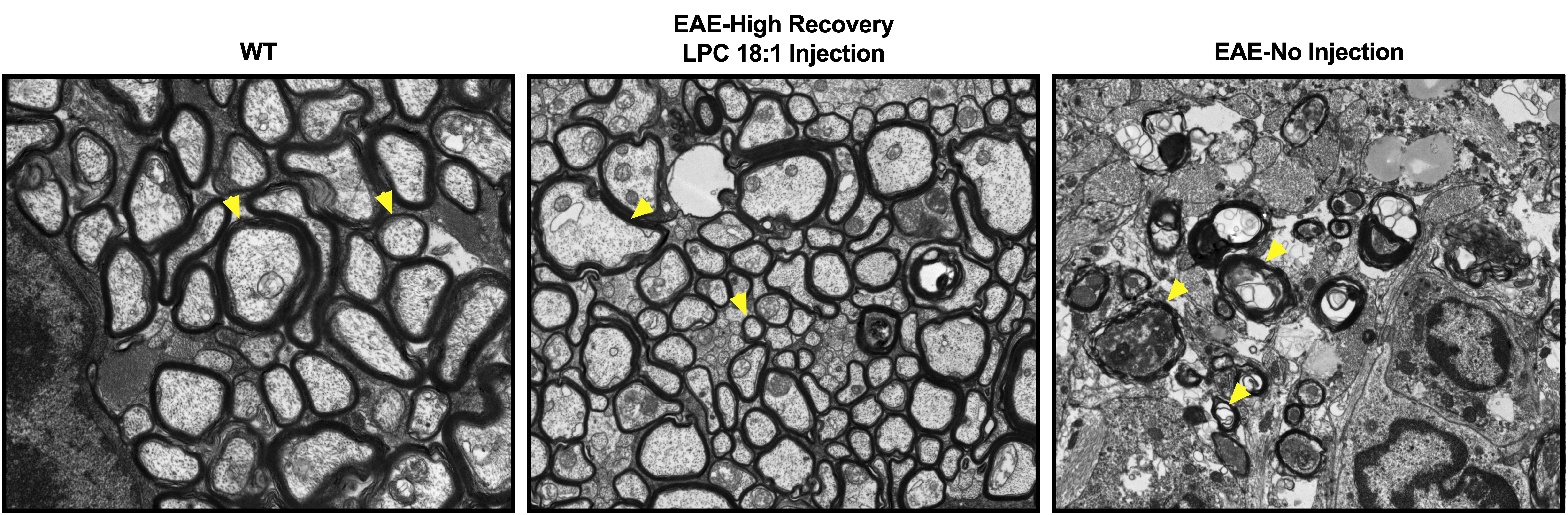

Supplement: Figure 2-3 — Electron microscopy of EAE optic nerves injected with LPC 18:1. Left, Representative coronal cross section for WT optic nerves (noninjected). Middle, Representative optic nerve for EAE mice in the high-recovery group (injected with LPC 18:1). Right, Representative optic nerve for EAE mice in the no-injection group. Yellow arrow points towards myelin sheath (dark rings). Myelin is observed to be compacted in the left and middle panels, whereas its less compact in the right panel. Download Figure 2-3, TIF file. [file enu-eN-NWR-0429-21-s04.tif]

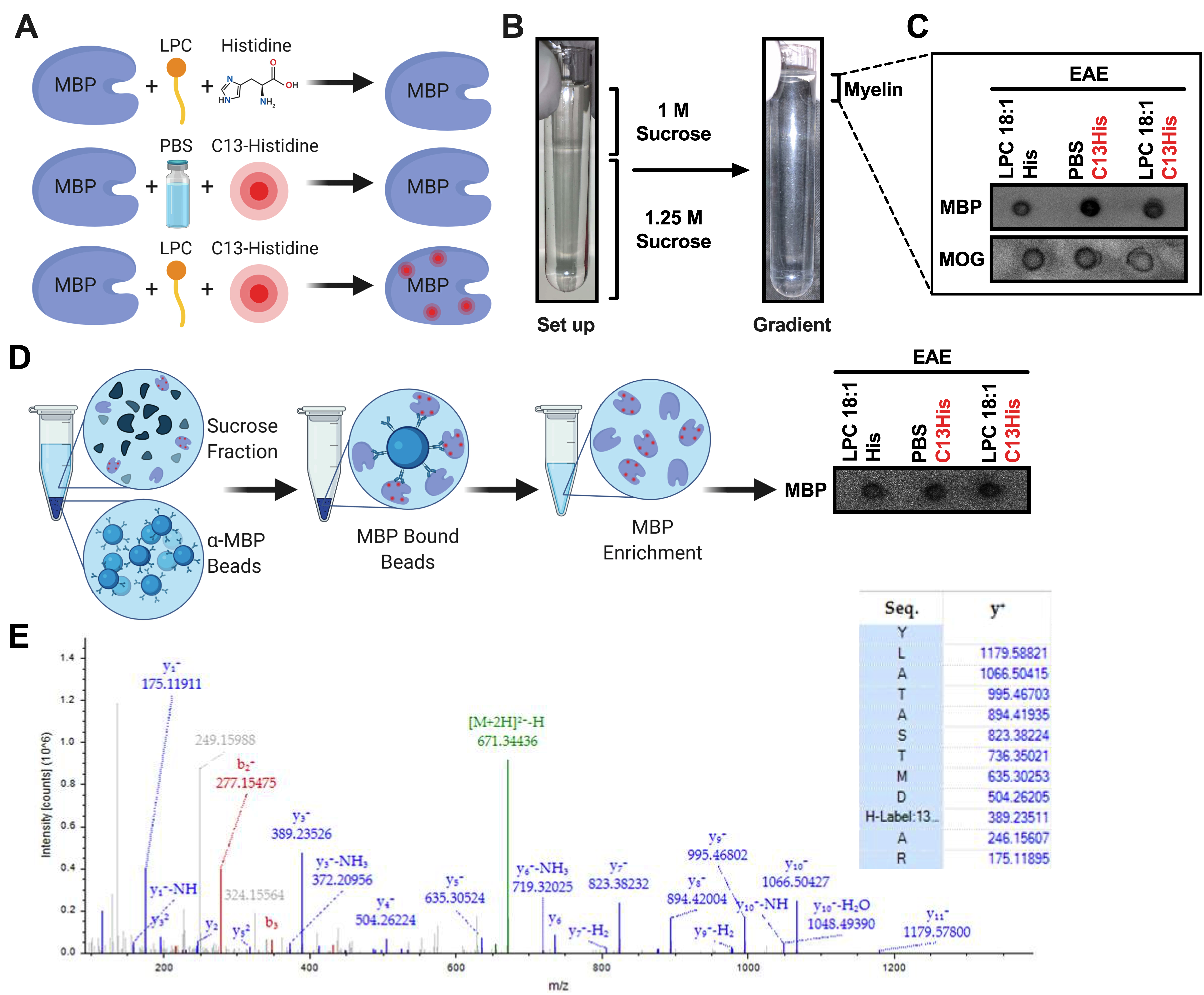

Supplement: Figure 3-1 — Isolation of myelin proteins from EAE optic nerves injected with isobaric C13-histidine. A, Schematic experimental setup for isobaric optic nerve injections. Only newly synthesized protein should incorporate isobaric C13-histidine in its sequence. B, Sucrose gradient ultracentrifugation of optic nerves (one optic nerve per tube). Myelin fraction is localized to the top of the gradient. C, Validation of the presence of myelin proteins in myelin fraction of the sucrose gradient ultracentrifugation. MBP: catalog #ab7349, Abcam; MOG: catalog #ab28766, Abcam. D, MBP enrichment for mass spectrometry analysis. MBP: catalog #ab7349, Abcam. E, Mass spectrometry spectra for demonstrating incorporation of C13-histidine into MBP peptide sequence. Download Figure 3-1, TIF file. [file enu-eN-NWR-0429-21-s05.tif]

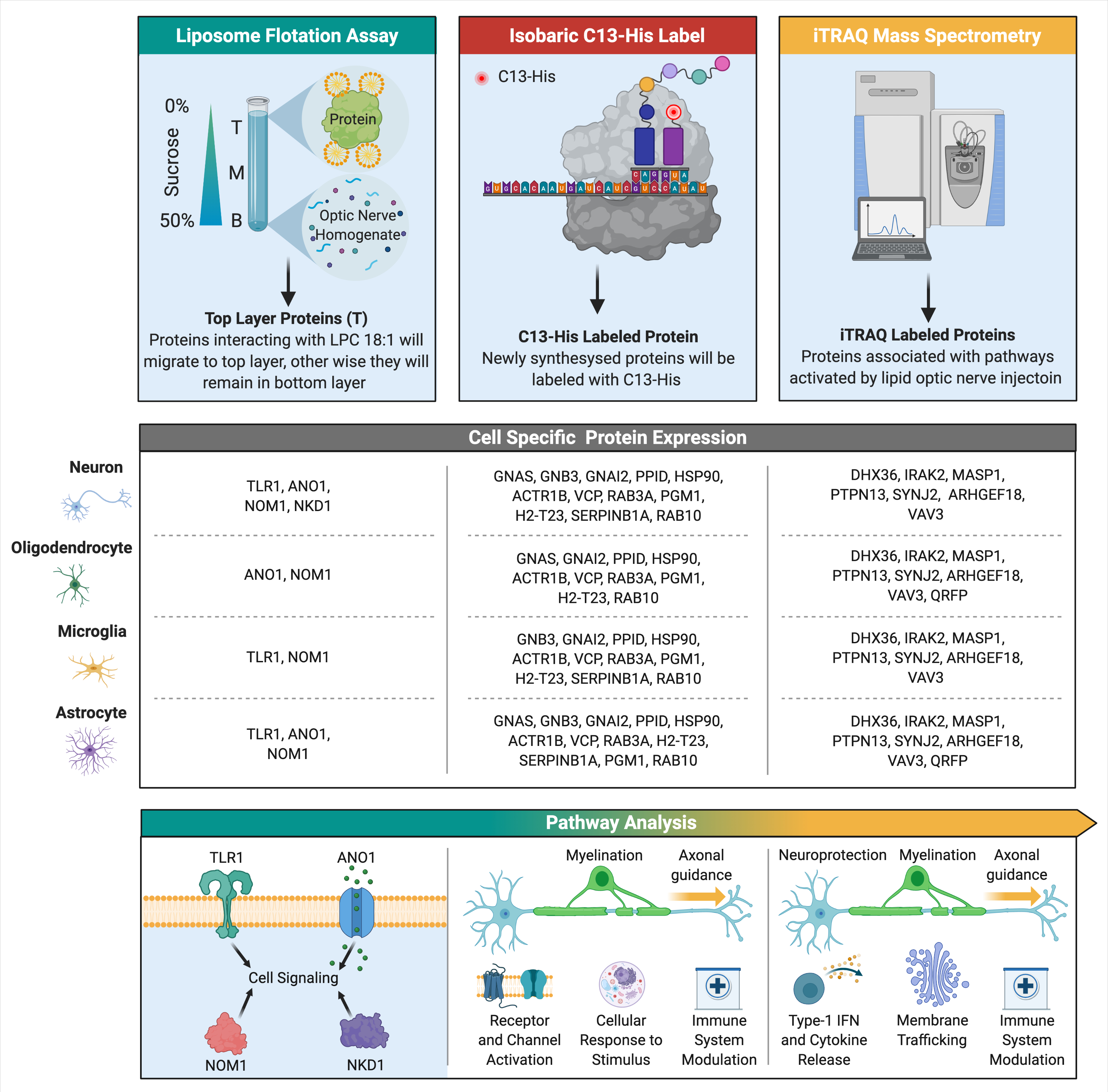

Supplement: Figure 3-2 — LPC 18:1 optic nerve injections promote myelination and neuroprotection. Proteins identified using the liposome flotation assay complex directly with micelles made from LPC 18:1. Proteins labeled during the isobaric C13-histidine experiment are proteins that are newly synthesized as a consequence of LPC 18:1 signaling. iTRAQ analysis identifies proteins that have a gradual increase in abundance in the following pattern: No-Injection → Low-Recovery → High-Recovery → Stay-Normal. Cell-specific protein expression was referenced using the EMBL-EBI Expression Atlas database. Reactome pathway analysis demonstrated protective and beneficial pathways being activated. Download Figure 3-2, TIF file. [file enu-eN-NWR-0429-21-s06.tif]

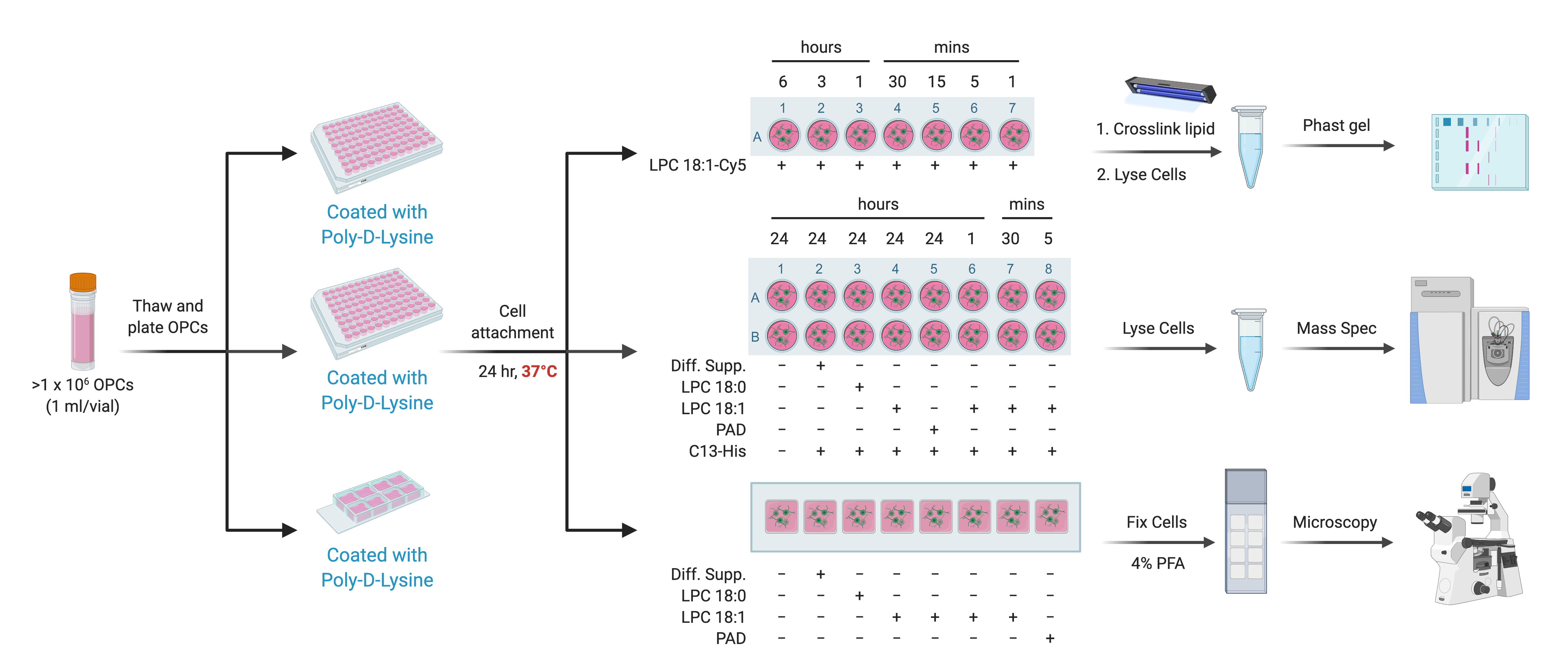

Supplement: Figure 4-1 — LPC 18:1 oligodendrocyte progenitor cell differentiation experimental setup. Experimental setup for rat OPC cultures. OPC were plated at 3 × 10^4^ cells per well/chamber (10 μl for 96-well plate and 30 μl for slide chamber). OPCs were incubated at 37°C for 24 h (incubated >24 h if cells were not fully attached). Three different experimental setups included the following: (1) plating and exposing OPCs to LPC 18:1-Cy5 (10 μm) followed by UV cross-linking, cell lysis, and PhastGel separation; (2) plating and exposing OPCs to differentiation supplement as a positive control, LPC 18:0 (10 μm), LPC 18:1 (10 μm), and PAD followed by cell lysis and mass spectrometry analysis. Only the treated groups included isobaric C13-histidine; and (3) plating and exposing OPCs to LPC 18:0 (10 μm), LPC 18:1 (10 μm), and PAD followed by fixation using 4% paraformaldehyde (PFA) and imagining. Download Figure 4-1, TIF file. [file enu-eN-NWR-0429-21-s10.tif]

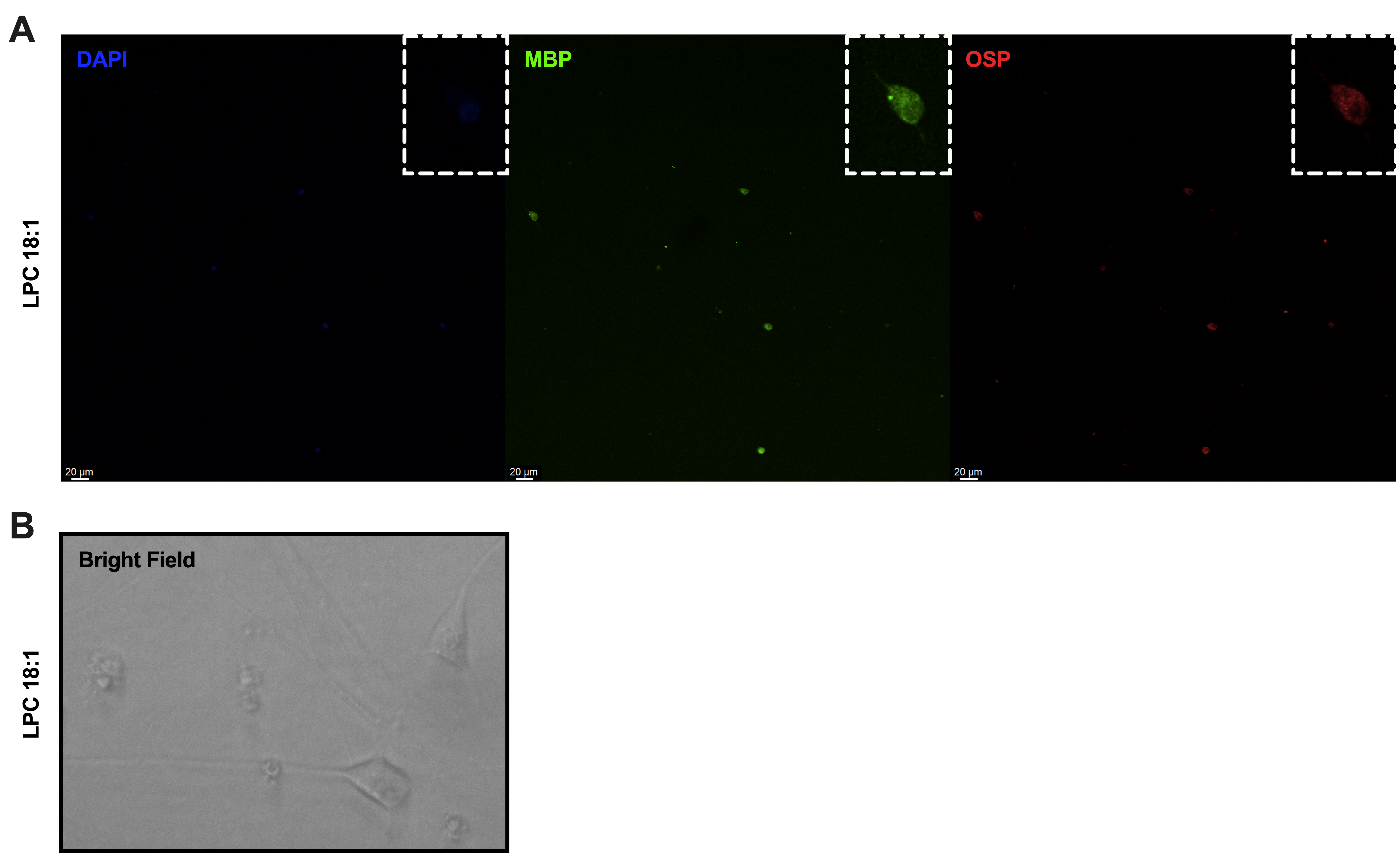

Supplement: Figure 4-2 — Rat OPCs treated with LPC 18:1. A, Immunohistochemistry of rat OPCs treated with LPC 18:1. MBP: catalog #ab7349, Abcam; OSP: catalog #ab7474, Abcam. Blue, DAPI; green, MBP; red, OSP. Scale bar, 25 μm. B, Rat OPC cell culture treated with LPC 18:1 (bright field). Download Figure 4-2, TIF file. [file enu-eN-NWR-0429-21-s11.tif]

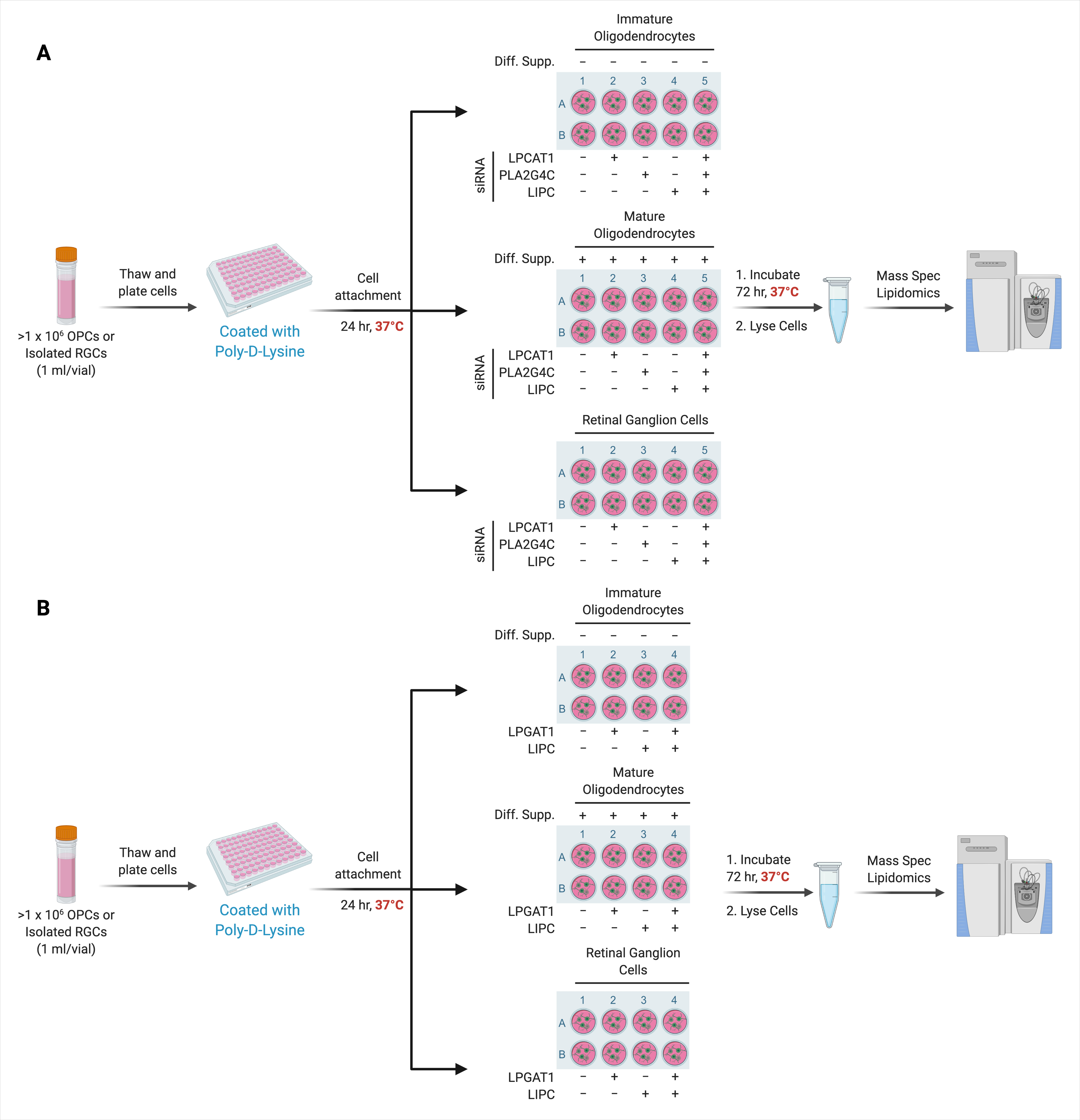

Supplement: Figure 4-3 — Lyso-lipid metabolic enzymes mediate levels of LPC 18:1 experimental setup. A, Experimental setup for silencing of LPC metabolic enzymes via siRNA. Rat OPCs and mouse RGCs were plated at 3 × 10^4^ cells/well/chamber (10 μl for 96-well plate and 30 μl for slide chamber). OPCs and RGCs were incubated at 37°C for 24 h (incubated >24 h if cells were not fully attached). OPCs were either differentiated into oligodendrocytes through the addition of differentiation supplement. OPCs, oligodendrocytes and RGCs were transfected with siRNAs for LPCAT1, PLA2G4C, and LIPC followed by cell lysis and mass spectrometry lipidomic analysis for LPC 18:1 levels. B, The same experimental setup as described above was implemented with the exception of cell transfection. Cells were transfected with an overexpression construct for LPGAT1 and LIPC. Cells were lysed and analyzed by mass spectrometry for levels of LPC 18:1. Download Figure 4-3, TIF file. [file enu-eN-NWR-0429-21-s12.tif]

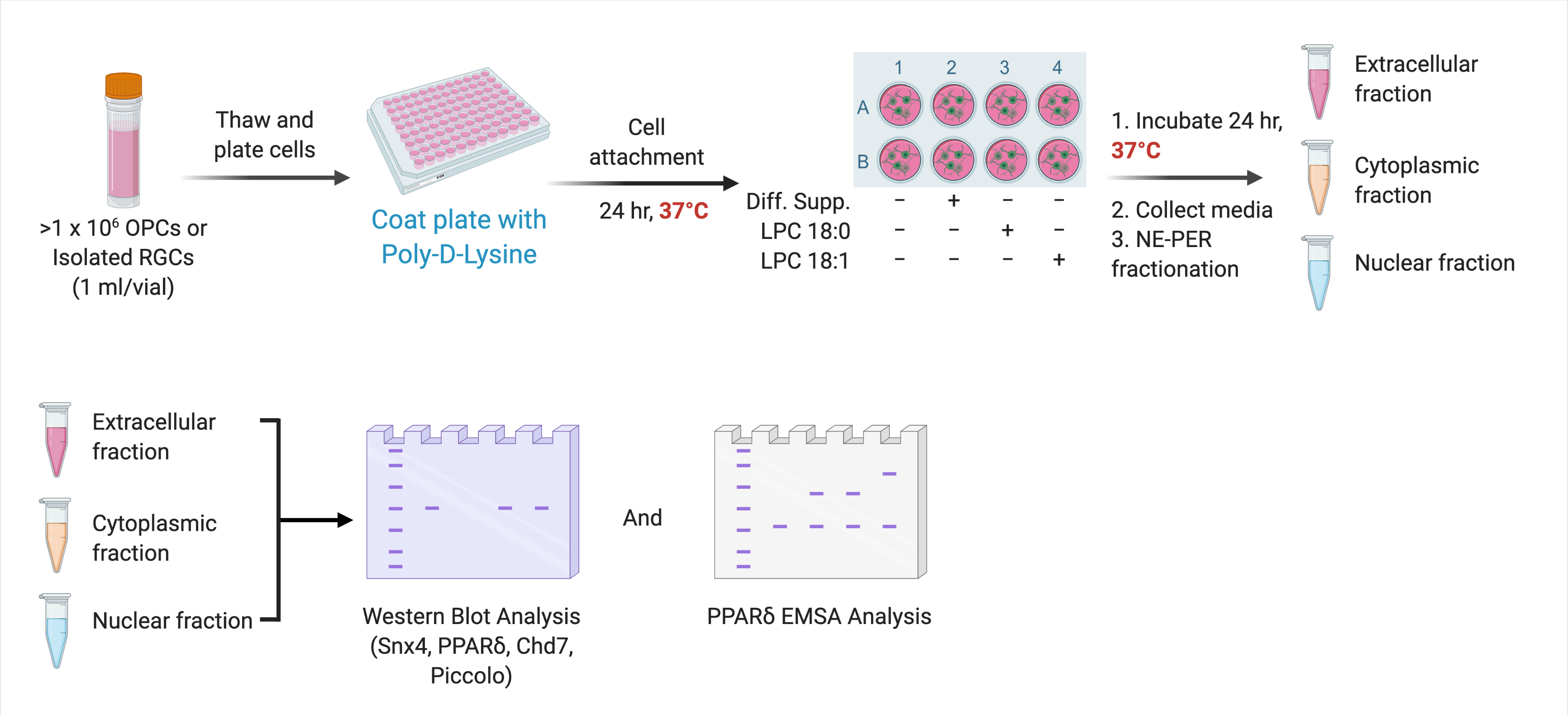

Supplement: Figure 4-4 — Cellular fractionation and identification of proteins of interest experimental setup. Experimental setup for rat OPC cultures. OPCs were plated at 3 × 10^4^ cells/well (10 μl for 96-well plate). OPCs were incubated at 37°C for 24 h (incubated >24 h if cells were not fully attached) followed by treatment with differential supplement control, LPC 18:0 (10 μm) or LPC 18:1 (10 μm). Cells were incubated for 24 h followed by media collection (extracellular fraction) and NE-PER cellular fractionation. Cellular fractions were analyzed using Western blot for the presence of proteins of interest (SNX4, PPAR-δ, CHD7, and PCLO). The nuclear fraction was further analyzed via electrophoretic mobility shift assay. Download Figure 4-4, TIF file. [file enu-eN-NWR-0429-21-s13.tif]

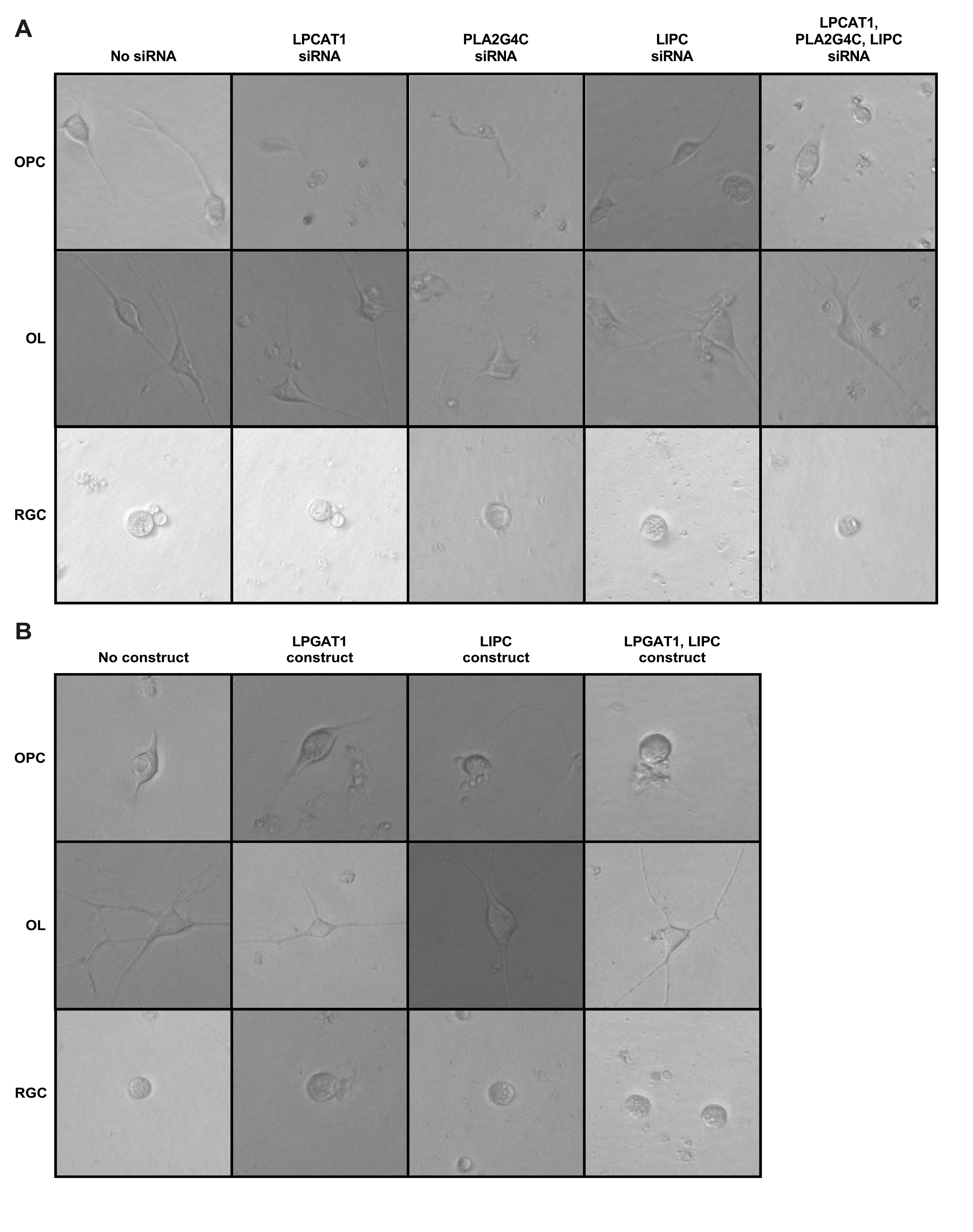

Supplement: Figure 4-5 — OPC cell culture with siRNA and overexpression construct. A, Representative cell culture morphology for rat OPCs treated with siRNA, rat oligodendrocytes (OL) treated with siRNA and mouse RGCs treated with siRNA. B, Representative cell culture morphology for rat OPCs treated with overexpression construct, rat oligodendrocytes (OLs) treated with overexpression construct and mouse RGCs treated with overexpression. OPC and OL cultures were validated using mRNA and protein analyses following published articles (Jäkel et al., 2019). Download Figure 4-5, TIF file. [file enu-eN-NWR-0429-21-s14.tif]

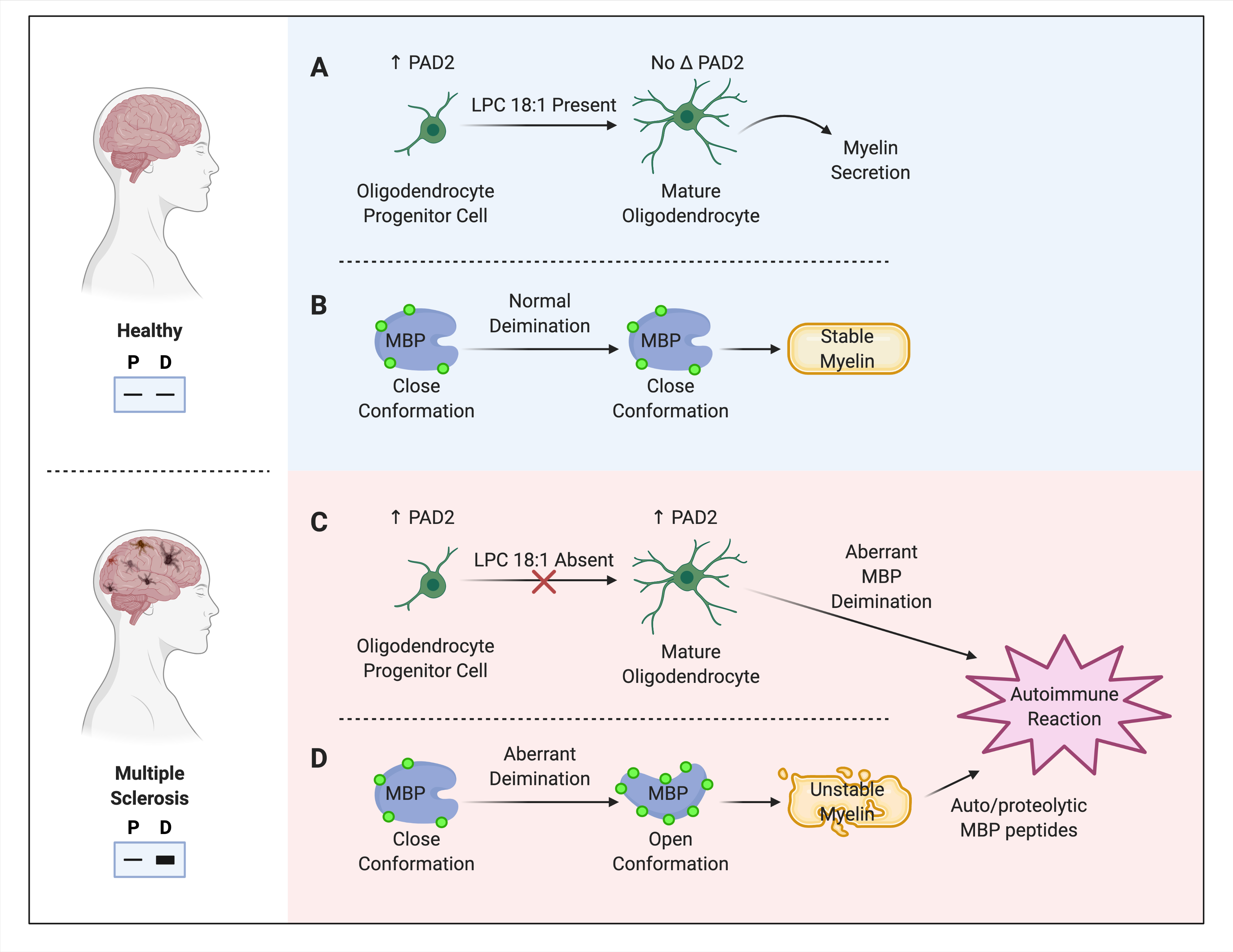

Supplement: Figure 4-6 — Proposed model for LPC 18:1-mediated immune response. Top left model depicts the levels of protein (P) and deimination (D) during healthy conditions. Both levels are equal. Bottom left model depicts the levels of P and D during multiple sclerosis. Protein levels are stable; however, the levels of deimination increase. A, Increase in deimination and the presence of LPC 18:1 permit the development of oligodendrocyte progenitor cells into mature oligodendrocytes. This process is permissible for myelination. B, During myelination, MBP maintains baseline levels of deimination and a close conformation. This close conformation allows for the formation of a stable compact myelin sheath. C, In the absence of LPC 18:1, oligodendrocyte progenitor cells cannot fully differentiate, which leads to an accumulation of aberrant hyperdeimination. Increased hyperdeimination on MBP is associated with priming of the immune system. D, Aberrant hyperdeimination of MBP is also associated with an open conformation, which makes MBP more susceptible to proteolysis. This increase in proteolysis depletes MBP from the myelin sheath, making it unstable and susceptible to demyelination. All of these events contribute to the autoimmune response observed in multiple sclerosis. Download Figure 4-6, TIF file. [file enu-eN-NWR-0429-21-s15.tif]
