# Supplementary material for: Lyso-Lipid-Induced Oligodendrocyte Maturation Underlies Restoration of Optic Nerve Function
Source: eNeuro. 2022 Jan 24;9(1):ENEURO.0429-21.2022. doi: 10.1523/ENEURO.0429-21.2022 (PMC8805197; doi:10.1523/ENEURO.0429-21.2022)
Supplement: Figure 3-3 — iTRAQ NI-LR-HR-SN. Download Figure 3-3, DOC file. [file enu-eN-NWR-0429-21-s07.doc]

| Figure 3-3. iTRAQ NI-LR-HR-SN | |  |
| --- | --- | --- |
| Pathway | Proteins | p-value |
| 1) Production of type I IFN and inflammatory cytokines  2) Lectin pathway of complement activation  3) Synthesis of PIPs at the plasma membrane and membrane trafficking  4) Promotes myelination, axon guidance and tight junction disassembly  5) Promotes neuroprotection  6) Mediate cell death and inflammation | 1) Dhx36, Irak2  2) Masp1  3) Ptpn13, Synj2  4) Arhgef18, Vav3  5) Qrfpr  6) Irak2 | 1) 0.004962689  2) 0.019624524  3) 0.029294749  4) 0.029592974  5) 0.03805237  6) 0.04362506 |
| iTRAQ analysis for proteins that are positively and negatively correlated with the following pattern: No-Injection – Low Recovery – High Recovery – Stay Normal (NI-LR-HR-SN). Pathway analysis was determined using the Reactome software version 75. | | |
