# Supplementary material for: Lyso-Lipid-Induced Oligodendrocyte Maturation Underlies Restoration of Optic Nerve Function
Source: eNeuro. 2022 Jan 24;9(1):ENEURO.0429-21.2022. doi: 10.1523/ENEURO.0429-21.2022 (PMC8805197; doi:10.1523/ENEURO.0429-21.2022)
Supplement: Figure 3-4 — Isobaric C13-histidine. Download Figure 3-4, DOC file. [file enu-eN-NWR-0429-21-s08.doc]

| Figure 3-4. Isobaric C13-Histidine | |  |
| --- | --- | --- |
| Pathway | Proteins | p-value |
| 1) Axonal guidance, myelination  2) Activation of GIRK channel, GABA receptor, Prostacyclin receptor, Thromboxane receptor  3) Immune system modulation  4) Cellular response to external stimuli | 1) Ezr, Dnm1, Cltc, Actb, Sptb, Hsp90ab1, Itgb1  2) Gnb2, Gnb3, Gnas, Gnai2  3) Dnm1, Capza1, Cltc, Actr1b, Vcp, Rab3a, Pgm1, H2-T23, Eef2, Actr1b, Serpinb1a, Hsp90ab1, Rab10  4) Hsp90ab1, Capza1, Stip1, Vcp, Hspa12a | 1) 2.83E-05  2) 0.00194405  3) 0.036384425  4) 0.006 |
| Pathway analysis of proteins that incorporated isobaric C13-Histidine during cell culture. Pathway analysis was determined using the Reactome software version 75. | | |
