# Supplementary material for: Lyso-Lipid-Induced Oligodendrocyte Maturation Underlies Restoration of Optic Nerve Function
Source: eNeuro. 2022 Jan 24;9(1):ENEURO.0429-21.2022. doi: 10.1523/ENEURO.0429-21.2022 (PMC8805197; doi:10.1523/ENEURO.0429-21.2022)
Supplement: Figure 3-5 — Proteins complexing with LPC 18:1 and LPC 18:0 micelles. Download Figure 3-5, DOC file. [file enu-eN-NWR-0429-21-s09.doc]

| Figure 3-5. Proteins complexing with LPC 18:1 and LPC 18:0 micelles | |
| --- | --- |
| LPC 18:1 | |
| UniProt Acc. # | Protein Name |
| Q9EPQ1  Q8BHY3  Q99MH6  Q3UFM5 | Toll-like receptor 1 (Tlr1)  Anoctamin-1 (Ano1)  Protein naked cuticle homolog 1 (Nkd1)  Nucleolar MIF4G domain-containing protein 1 (Nom1) |
| LPC 18:0 | |
| UniProt Acc. # | Protein Name |
| P04370  Q91XV3  P60710  P05213  P68369  P62874  Q6PIC6  Q7TMM9  P68372  Q8VDN2 | Myelin basic protein (MBP)  Brain acid soluble protein 1 (Basp1)  Actin, cytoplasmic 1 (Actb)  Tubulin alpha-1B chain (Tuba1b)  Tubulin alpha-1A chain (Tuba1a)  Guanine nucleotide-binding protein G(I)/G(S)/G(T) subunit beta-1 (Gnb1)  Sodium/potassium-transporting ATPase subunit alpha-3 (Atp1a3)  Tubulin beta-2A chain (Tubb2a)  Tubulin beta-4B chain (Tubb4b)  Sodium/potassium-transporting ATPase subunit alpha-1 (Atp1a1) |
| Proteins identified in the top fraction of the liposome flotation assay (LFA) that are complexing with LPC 18:1 and LPC 18:0. Mass spectrometry analysis of only high confidence proteins. | |
